# Polymeric Approaches to Reduce Tissue Responses Against Devices Applied for Islet-Cell Encapsulation

**DOI:** 10.3389/fbioe.2019.00134

**Published:** 2019-06-04

**Authors:** Shuixan Hu, Paul de Vos

**Affiliations:** Division of Medical Biology, Department of Pathology and Medical Biology, Immunoendocrinology, University of Groningen and University Medical Center Groningen, Groningen, Netherlands

**Keywords:** transplantation, islet, encapsulation, polymer, host response, surface properties, biocompatibility

## Abstract

Immunoisolation of pancreatic islets is a technology in which islets are encapsulated in semipermeable but immunoprotective polymeric membranes. The technology allows for successful transplantation of insulin-producing cells in the absence of immunosuppression. Different approaches of immunoisolation are currently under development. These approaches involve intravascular devices that are connected to the bloodstream and extravascular devices that can be distinguished in micro- and macrocapsules and are usually implanted in the peritoneal cavity or under the skin. The technology has been subject of intense fundamental research in the past decade. It has co-evolved with novel replenishable cell sources for cure of diseases such as Type 1 Diabetes Mellitus that need to be protected for the host immune system. Although the devices have shown significant success in animal models and even in human safety studies most technologies still suffer from undesired tissue responses in the host. Here we review the past and current approaches to modulate and reduce tissue responses against extravascular cell-containing micro- and macrocapsules with a focus on rational choices for polymer (combinations). Choices for polymers but also choices for crosslinking agents that induce more stable and biocompatible capsules are discussed. Combining beneficial properties of molecules in diblock polymers or application of these molecules or other anti-biofouling molecules have been reviewed. Emerging are also the principles of polymer brushes that prevent protein and cell-adhesion. Recently also immunomodulating biomaterials that bind to specific immune receptors have entered the field. Several natural and synthetic polymers and even combinations of these polymers have demonstrated significant improvement in outcomes of encapsulated grafts. Adequate polymeric surface properties have been shown to be essential but how the surface should be composed to avoid host responses remains to be identified. Current insight is that optimal biocompatible devices can be created which raises optimism that immunoisolating devices can be created that allows for long term survival of encapsulated replenishable insulin-producing cell sources for treatment of Type 1 Diabetes Mellitus.

## Introduction

Type one diabetes mellitus (T1D) impacts 1.25 million individuals in the US alone and is associated with an annual health care cost of $9.8 billion (American Diabetes Association, 2018). These costs can be reduced by tight regulation of the blood glucose levels such as can be done with allogeneic transplantation of pancreatic islets. Up to now these islets are obtained from cadaveric donors that regulate glucose levels from a minute-to-minute level (Choby, 2017). This replaces insulin injections and prevents regular hypoglycemic events and thereby contributes to improved quality of life. The mandatory use of immunosuppression to prevent graft rejection is unfortunately an obstacle for large scale application. Application may be facilitated with effective encapsulation technologies for immunoprotection of islets that prevent graft rejection and autoimmune destruction of islets (Barkai et al., 2016). To generate immunoisolative membranes, several materials have been explored but an ongoing challenge remains prevention of too strong tissue responses that might lead to graft failure (Paredes-Juárez et al., 2014b). The tissue responses might manifest *in vivo* as immune cell adhesion and fibrotic overgrowth on the surface of micro- or macrocapsules but also strong responses in the immediate vicinity of the capsules might lead to cytokine production and death of islet-cells (de Vos, 2017; Krishnan et al., 2017). Here we review current and past approaches in which polymer engineering has been applied to improve biocompatibility of natural and synthetic polymers applied for islet micro- or macroencapsulation.

### Need for Islet Transplantation in T1D

In T1D insulin-producing pancreatic β cells are destroyed by a specific autoimmune reaction resulting from a complex of environmental and genetic factors (Atkinson et al., 2014). This autoimmune destruction is irreversible, which implies lifelong insulin administration by injections to regulate homeostasis of blood glucose (Hirsch, 2009). Although this therapy is life-saving, it has a major impact on the quality of life of patients. Patients need to be taught to self-monitoring blood sugars and to adjust insulin dosing according to daily needs. Despite this intensive way of regulating glucose levels, it cannot regulate blood glucose on a minute-by-minute basis. As a consequence, of this lack of precise regulation diabetic complications may develop such as retinopathy, neuropathy, and cardiovascular disease (Choby, 2017). Also, intensive insulin therapy holds the threat of regular hypoglycemic episodes which might eventually lead to hypoglycemic unawareness (Bragd et al., 2003). Better and more precise regulation of glucose levels is highly needed to prevent diabetic complications, and for improving patient's life quality.

Ever since the groundbreaking publication of the Edmonton protocol (Shapiro et al., 2000), which reported insulin-independence in seven recipients after an average of 12 months, pancreatic-islet transplantation provides an alternative strategy to restore physiological insulin-responses to plasma glucose changes (Berney et al., 2009). Since that time 1,086 patients were transplanted with islets according to the Collaborative Islet Transplant Registry (CITR) 10th Annual Report (Collaborative Islet Transplant Registry, 2017). These patients all have a complete absence of hypoglycemia, in many cases remain insulin independent and most of them experienced an improved quality of life (Ryan et al., 2002, 2005). Despite these successes, islet transplantation is not yet a widely applied treatment for T1DM. The reason for that is the mandatory use of life-long immunosuppression of the patient to prevent graft rejection (Berney et al., 2009). Immunosuppression is associated with increased risk for serious infections and cancer (Dantal and Soulillou, 2005), as well as associated with metabolic disorders and toxicity for kidneys (Ekberg et al., 2009). Immunosuppression is therefore not considered to be an acceptable alternative for insulin therapy (Ricordi and Strom, 2004).

## Islets Encapsulation Technology

An advantage of islet-transplantation over whole pancreas transplantation is that islets are clumps of cells that can be packed in immunoisolating membranes. Immunoisolation is a technology that potentially allows for transplantation of islets in the absence of life long immunosuppression. Within this technology islets are encapsulated inside semi-permeable membranes that can isolate islet grafts from immune cells and antibodies of recipients while allowing ingress of nutrients, oxygen and glucose, and egress of insulin (de Vos et al., 2010). In the past three decades, three major categories of encapsulation approaches were studied for islet immunoisolation. These include intravascular macrocapsules, extravascular macrocapsules, and extravascular microcapsules (Teramura and Iwata, 2010; O'Sullivan et al., 2011). Intravascular devices are connected to the bloodstream which implies fast correction of changes in blood-glucose levels due to faster exchange of glucose and insulin (Prochorov et al., 2008). However, its clinical application was and is limited by high risks for thrombosis and infections, and the demand for major surgery for implantation. Although some groups still publish novel approaches for intravascular devices that are associated with less risks (Prochorov et al., 2008; Gmyr et al., 2017), the majority of research papers in the past decade focus on extravascular devices. Extravascular devices are therefore the major focus of this review.

**Figure 1:**
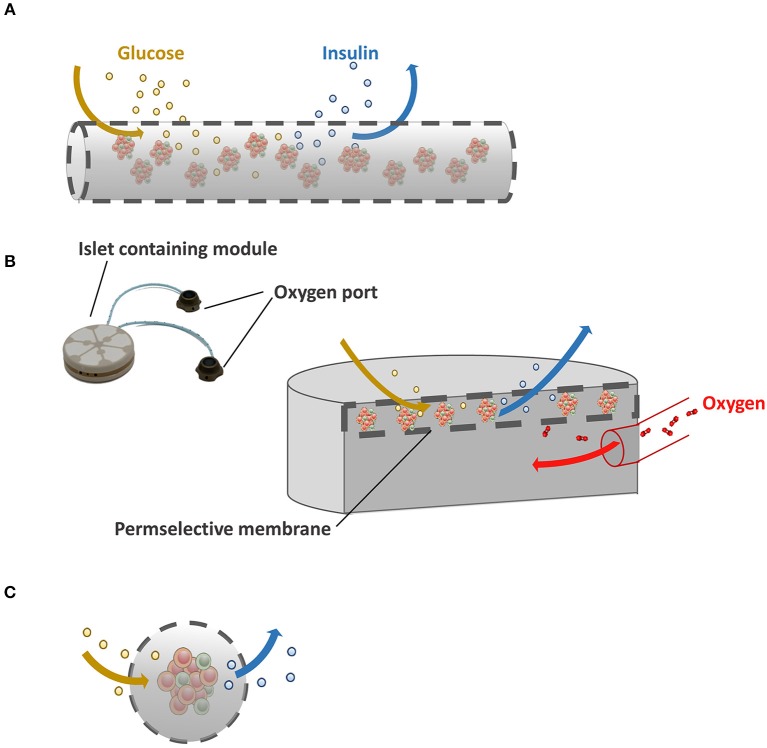
Immunoisolating devices. **(A)** In macrocapsules, groups of islets are encapsulated in a selectively permeable membrane. Because of the unfavorable volume to surface ratio in macroencapsules insufficient supply of nutrients such as oxygen is a major issue. **(B)** Schematic illustration of Beta-O2 device. Beta-O2 is equipped with a refillable oxygen chamber that allows the diffusion of oxygen to the islet-containing chamber. **(C)** Schematic illustration of microcapsules with a better surface to volume ratio than macrocapsules which facilitates ingress of oxygen and glucose and egress of insulin.

Extravascular devices can be distinguished into macro- and microcapsules. Macrodevices contain groups of islets inside the membrane (Figure 1A). The technique is rather simple in concept. Groups of islets are encapsulated in the devices and implanted either subcutaneously or intraperitoneally without direct connection to the blood stream. Within days blood vessels grow toward the surface for mandatory nutrient supply, but also to exchange glucose and insulin. A major issue in the field of macrocapsules, however, is the unfavorable surface to volume ratio (Orive et al., 2018). As a consequence, diffusion of essential nutrients such as oxygen is slow and islets inside the capsules compete for these nutrients. Because of this there is a limitation in seeding density that almost never exceeds 5–10% of the volume of the devices (Lacy et al., 1991).

A promising solution for this diffusion issue is the so-called Beta-O2 device (Figure 1B). Beta-O2 is a bioartificial pancreatic device, which is implanted under the skin or into the pre-peritoneal cavity with minimal surgery. The Beta-O2 device consists of two modules. A chamber is connected with an oxygen port that allows infusion of gas into a chamber by an injector that is operated manually. The other module is the islet graft containing capsule which is surrounded by a perm-selective membrane consisting of three layers, i.e., a polytetrafluoroethylene, a high mannuronic acid alginate gel, and a silicon rubber (Barkai et al., 2016). The multilayer membrane allows free diffusion of oxygen, glucose, and insulin and forms an effective immunoisolating membrane (Ludwig et al., 2010). Due to the presence of an oxygen supply module more islets can be encapsulated into a predefined volume without hypoxia. In the original concept of the Beta-O2 device, 2400 IEQ/device were loaded at a surface density of 1,000 IEQ/cm^2^ with a refueling every 2 h with atmospheric air (Barkai et al., 2013). With this device diabetic rat recipients maintained normoglycemia through up to 240 days which was the end point of the experiment. Also, efficacy of this approach was demonstrated in a large animal model, i.e., mini-pigs. The device with two separated islet modules attached to a gas chamber containing 6,730 ± 475 rat IEQ/kg body weight (BW) was introduced in diabetic mini-pigs. The rat islets induced normoglycemia up to 75 days without immunosuppression demonstrating efficacy and safety as well as the ability to use xenogeneic approaches with the device in larger mammals (Neufeld et al., 2013). Efficacy of xenogeneic porcine islets was recently also shown in a nonhuman primate model with T1D with 20,000 islets/kg BW (Ludwig et al., 2017). The device induced a persistent stable glycemic control even during a stepwise reduction in daily exogenous insulin dose up to 190 days after which the devices were explanted (Ludwig et al., 2017). Upon retrieval, a strongly vascularized fibrous capsule was observed around the device that according to the authors facilitates the exchange of substances in and out of the device (Ludwig et al., 2017).

Microcapsules in contrast to macrocapsules do suffer less from diffusion issues as they have a very optimal volume to surface ratio (Figure 1C). Other advantages are that when a minority of microcapsules are suffering from cell adhesion due to local imperfections (de Vos et al., 1996a; De Vos et al., 1996b) the grafts will not immediately fail while such a response is more deleterious for macrodevices. Additionally, microcapsules are mechanically stable and encapsulation can be done with nontoxic molecules and reagents (Bhujbal et al., 2014a). The majority of encapsulation approaches use alginate as core material followed by poly-amine thin coating to provide immunoprotection or to enhance mechanical stability (Kendall and Opara, 2017). To enhance biocompatibility many different alginates with a large variation of chemical modifications have been tested. In one of the studies, 744 alginate analogs were tested, which revealed 200 analogs associated with lower immune cell activation compared to the others (Vegas et al., 2016a). The evaluation of alginate analogs in both rodents and non-human primates identified three analogs that showed little presence of macrophages and fibroblasts on the capsule surface demonstrating that alginates are biocompatible in the correct chemical structure (Vegas et al., 2016a; Bochenek et al., 2018). A challenge in this area is however to identify and document the relationships between the surface properties and biocompatibility because even the microcapsules tested in the studies had different surface properties (Vegas et al., 2016a) and provoked different degrees of tissue responses.

Although the large surface to volume ratio of microcapsules facilitates oxygen and nutrient diffusion, the optimal size of capsules to prevent tissue responses has recently become subject of debate (Veiseh et al., 2015; de Vos, 2017). It was reported that microcapsules with a diameter of 500 μm induced significantly more macrophage and fibroblast adhesion on the surface than capsules of 1,800 μm (Veiseh et al., 2015). Remarkably, we and others using microcapsules in the 0.5 mm range (Orive et al., 2006; de Vos et al., 2009; Hall et al., 2011; Paredes-Juarez et al., 2013) never observed these responses. A possible explanation form this (de Vos, 2017) might be a variations in the level of alginate purityused by the different groups (Paredes-Juarez et al., 2013, 2014a; Paredes-Juárez et al., 2014b). Veiseh et al did not apply alginates that were purified and were free of endotoxins (Veiseh et al., 2015). These endotoxins will diffuse after capsule formation to the surface. As smaller capsules have a higher surface to volume ratio than larger capsules, more immune stimulatory endotoxins will be present on the surface of the smaller capsules, leading to stronger tissue responses (Paredes-Juarez et al., 2013, 2014a; Paredes-Juárez et al., 2014b; de Vos, 2017). It is well known that alginate which is not sufficiently purified may provoke stronger tissue responses than purified alginates (Liu et al., 2011; Fang et al., 2017b). We but also others (Tomei et al., 2014; Manzoli et al., 2017, 2018; Buchwald et al., 2018a) do not see severe responses against small capsules and also recognize that larger diameters for capsules also implies lower oxygen supply to the islets (Tomei et al., 2014; Manzoli et al., 2017; Buchwald et al., 2018a; Komatsu et al., 2018; Tomei, 2018) which unfortunately is not discussed in the Veisah study (Veiseh et al., 2015). For this reason, we prefer and keep on working on smaller capsules (Spasojevic et al., 2014a; Paredes-Juarez et al., 2015; Llacua et al., 2018a,c) which will be further discusses in the next sections.

As mentioned above a major advantage of encapsulation is the possibility to use cells from non-human sources or a replenishable cell source from animal or human origin. World-wide there is a huge gap between supply and demand for cadaveric pancreata (Robertson, 2004; Bruni et al., 2014). This might be solved by using stem cell-derived insulin-producing cells or by using islets obtained from animals (Ekser et al., 2015). Encapsulation and protection from the recipients' immune system may facilitate clinical use of these cell sources. Due to significant progress in the field of stem-cell research and creation of a replenishable insulin-producing cell source, fundamental research toward better capsule formulations has revisited. Several groups report that encapsulated porcine islets, which is considered to be a replenishable insulin-producing cell source, successfully survived in non-human primates for over 6 months with both microencapsulation (Dufrane et al., 2006) and macroencapsulation (Dufrane et al., 2006) approaches. Another study with microencapsulated porcine islets reported up to 70 days survival in non-human primates which might be improved by enhancing oxygen supply (Safley et al., 2018). Successes also have been shown in human patients transplanted with microencapsulated porcine islets (Omami et al., 2017). A clinical study has reported improved HbA1c levels and reduced hypoglycemic episodes for more than 600 days (Matsumoto et al., 2016). Living Cell Technologies has performed a larger clinical study using Diabecell^®^, which is a commercial microencapsulated porcine islet graft which in humans resulted in a reduction in exogenous insulin use (Tan, 2010; Hillberg et al., 2013). Also, with stem-cells the usefulness of encapsulation technologies has been demonstrated. Pagliuca et al. (2014) transplanted alginate microencapsulated glucose-responsive stem-cell-derived β cells without any immunosuppression into T1D mice models which induced normoglycemia until their removal at 174 days after implantation (Vegas et al., 2016b). More recently, the maturation of human stem-cell-derived β cells was stimulated by forming islet-sized enriched β-clusters that responded to glucose stimulation as early as 3 days after transplant (Nair et al., 2019). This, however, is not the only development in replenishable cell sources in which cell-encapsulation is instrumental. Genome-editing techniques have been creating a novel field that might lead to new insulin-producing cell sources (Cooper et al., 2016).

Despite its revisiting and promising application, cell encapsulation in extravascular systems still suffer from a common issue which is host responses against the capsules. These responses might ultimately lead to adhesion of inflammatory cells, fibroblast, collagen deposits that interfere with nutrition of the cells in the devices (Krishnan et al., 2017). Some groups report more and stronger host responses than others (Orive et al., 2018) with seemingly similar approaches. In this review we discuss progress made in the field and novel approaches to reduce or delete these responses on extravascular devices. This involves choice of type of polymer, the absence of proinflammatory residues or contaminants in the devices or polymers, insights in chemical conformation of surfaces to reduce host responses but also novel approaches for biofouling or immunomodulating biomaterials and application of polymers that form polymer brushes. In addition, we discuss possible beneficial effects of local release of immunomodulation molecules or inclusion and/or co-encapsulation of immunomodulatory cells.

## Attenuate Host Responses by Rational Choices for Polymers

The original promise of the islet encapsulation technology is to hide islets from the host immune system and to make them untouchable (de Vos and Marchetti, 2002). This is still the basis of many membranes that have been developed over the past decade (Paredes-Juarez et al., 2013; Paredes-Juárez et al., 2014b; Paredes-Juarez et al., 2015; Llacua et al., 2016). Another pertinent aim is to use and design encapsulation materials that are biocompatibility and are having a permeability that guarantees protection against larger immune mediators such as immunoglobulins and complement factors but at the same time allowing exchange of essential nutrients in and out of capsules (Grace et al., 2016). The polymers that have been tested are derived from both natural sources or synthetic. There are different classes of natural polymers i.e., polysaccharides, polypeptide, and polynucleotides, of which polysaccharides are the most commonly used in cell encapsulation. They offer several advantages over the other two natural sources. They can provide cells with a membrane in a relatively mild fashion and generally without application of toxic solvents (de Vos et al., 2014). Furthermore, the majority of polysaccharides form hydrogels that are as flexible as natural tissue, mechanically stable (Li, 1998), and reportedly associated with minor host responses (Cieslinski and David Humes, 1994). Synthetic polymers are also widely investigated. Theoretically synthetic polymers can be reproducibly be produced without batch-to-batch variation. Another relevant advantage is that synthetic polymers can be tailor-made to improve biocompatibility or to induce other desired properties (Miura et al., 2006; Najjar et al., 2015; Pham et al., 2018).

### Alginate

**Figure 2:**
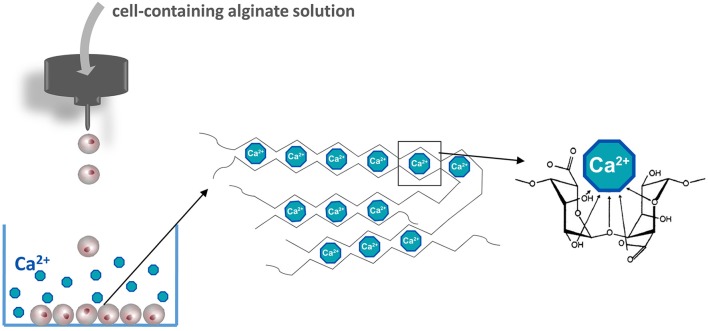
Manufacturing islet-containing alginate-based microcapsules. Islets are suspended in an alginate solution solved in a balanced physiological salt solution in the absence of calcium. Alginate containing islet droplets are formed by an air- or electrostatic driven droplet generator. Droplets are collected in a CaCl_2_ solution to form microcapsules. The basis of the gel formation is calcium crosslinking constitutive alginate molecules according to the egg-box model.

The most commonly applied and detailed studied polymer in encapsulation is alginate and applied in both macro- and microencapsulation approaches (Wang et al., 2011; Cañibano-Hernández et al., 2019). Alginate can be extracted from several organisms including *Azotobacter vinelandii*, several *Pseudomonas* species, and a variety of algae (Wee and Gombotz, 1998). Alginate is a natural anionic linear polysaccharide consisting of 1,4′-linked β-D-mannuronic acid (M) and α-L-guluronic acid (G) in different sequences or blocks, namely G-G blocks, G-M blocks, and M-M blocks (de Vos et al., 2014). The ratio and molecular weight of the blocks depends on the applied natural raw material for alginate extraction and is used to form capsules with different physical and chemical properties (Ostgaard et al., 1993; de Vos et al., 2014). Alginate capsules are usually formed by collecting cell-containing alginate droplets in a solution with a high concentration of cations. The cations in the solution bind to uronic acid blocks in alginate according a so-called egg-box model (Figure 2) (Li et al., 2007). The pliability and rigidity of alginate capsules depends on both the type of alginate and type of cation applied. Ca^2+^, Sr^2+^, and Ba^2+^ are having a high affinity and are in the concentration and duration of exposure not toxic for cells (Stokke et al., 1991). Gels generated from alginates with a high guluronic acid (High-G) content also form stronger gels (Uludag et al., 2000; de Vos et al., 2004; Bhujbal et al., 2014a). It was reported that the proinflammatory properties of alginate also depends on alginate types (Grace et al., 2016). Intermediate-G alginate provoked a lower immune response than low- and high-G alginate (Paredes-Juarez et al., 2013). This however can be changed by varying the cation types. Eg using barium instead of calcium in high-M alginates results in stable and biocompatible capsules. Barium in contrast to calcium can bind to both G-G and M-M and produces capsules with completely different properties. Duvivier-Kali et al. demonstrated with this approach survival of islet grafts in diabetic BALB/c and NOD mice for more than 350 days (Duvivier-Kali et al., 2001).

### Other Natural Polymers

In addition to alginate, there are many other natural polymers used in encapsulation, which have received less attention than alginate but have shown some success. These include agarose, chitosan, cellulose, and collagen (de Vos et al., 2014).

Agarose is produced from agar and associated with minimal immune responses (Fernández-Cossío et al., 2007; Takemoto et al., 2015). Some successes have been shown in diabetic dogs with allogeneic islets in agarose microcapsules inducing normoglycemia for up to 49 days without significant accumulation of inflammatory cells and fibroblasts around the capsules (Tashiro et al., 1997). In diabetic Balb/c mice agarose microencapsulated mouse islets induced normoglycemia for up to 56 days without inflammatory cell infiltration (Agudelo et al., 2009). Also, agarose macrocapsules have been tested in diabetic mice (Iwata et al., 1994) and pancreatectomized dogs (Gazda et al., 2014). The main challenge with agarose is to create a gel with sufficient immunoprotection as it does not block diffusion of cytotoxic immunoglobulin G (IgG) (Iwata et al., 1992a,b). In principle, the immunoprotective properties of agarose gels are determined by the concentration of agar solution to form perm-selective membranes. Usually 5% agarose is used to generate immunoprotective capsules (Kobayashi et al., 2003). However, to enhance immunoprotection in *in vivo* studies, the agarose concentration was raised to 7.5–10% (Iwata et al., 1994). Another approach to enhance immunoprotection has been coating of agarose microcapsules with poly-acrylamide, which successfully prevented the entry of antibodies but provoked major host responses (Dupuy et al., 1990). To overcome the host responses more complex three layer agarose-based immunoisolation systems were introduced (Tun et al., 1996). To improve immunoprotection and mechanical stability, 5% polystyrene sulfonic acid (PSSa) was added together with 5% agarose to form the core of microcapsules. A polybrene layer coating was applied to prevent the leakage of PSSa that may stimulate host responses. Another layer of carboxylmethyl cellulose as the outermost shell offered biocompatibility of microcapsules (Tun et al., 1996). In addition to fine tuning permeability to enhance immunoprotection, researchers also investigated the possibility to combine local immunosuppression by co-encapsulating SEK-1005. SEK-1005 is an anti-inflammatory agent (Kuriyama et al., 2000). The rod was explanted 10 days after implantation leaving a subcutaneous transplant site that was surrounded by highly vascularized granulomatous tissue (Kuwabara et al., 2018). Islet transplanted in the site survived more than 100 days without immunosuppression owning to regulatory T cells in the granulomatous tissue that regulated immune reactions against islet grafts (Takemoto et al., 2015).

Also chitosan has been proposed as alternative for alginate. Several groups have shown success with chitosan as a coating layer for alginate-based microcapsule to reduce pericapsular fibrosis (Yang et al., 2016). Chitosan-alginate complexes have been suggested to improve long-term mechanical stability (Baruch and Machluf, 2006). However, the application of chitosan in islet encapsulation is somewhat limited due to low solubility of chitosan under physiological pH (Kubota et al., 2000; Ruel-Gariépy et al., 2002; Yang et al., 2010). PH values as low as four are needed to solve the polymer. Islets are very sensitive for low pH. Significant attempts have been made to modify chitosan as such that it is soluble under more physiological pH. Novel water-soluble chitosan derivatives have been developed (Sobol et al., 2013) that can be dissolved at pH 7.0. These novel formulations are obtained from oligochitosan and different aliphatic amines. When applied as membrane for alginate/calcium beads, no negative effects were observed (Sobol et al., 2013). Another study focusing on chitosan derivatives synthesized methacrylated glycol chitosan (MGC) in a saline solution at pH 9. These MGC membranes on the outside of alginate capsules enhanced mechanical stability and were associated with less fibroblast overgrowth than alginate/poly-L-ornithine/alginate capsules (Hillberg et al., 2015). Another approach to generate chitosan hydrogel that allow capsule formation at physiological pH values is adding glycerol 2-phosphate disodium salt hydrate into acetic chitosan solution (Yang et al., 2010). Rat islets macroencapsulated in this hydrogel reversed hyperglycemia in diabetic mice with a progressive increase in body weight as a consequence (Yang et al., 2010).

Cellulose is also proposed for cell encapsulation but a poorly soluble polysaccharide and has been chemically modified to hydroxypropyl cellulose (Heng and Wan, 1997), carboxymethyl cellulose (Tun et al., 1996), and ethylcellulose (Wandrey et al., 2010) for better solubility facilitating application in cell-encapsulation processes. Cellulose has been applied as encapsulation material with rat (Wang et al., 1997), porcine (Schaffellner et al., 2005) and mouse islets (Risbud et al., 2003). A pertinent issue with cellulose derivates is controversies about its biocompatibility. Some groups report absence of host reactions to cellulose-based capsules (Pelegrin et al., 1998; Schneider et al., 2001), whereas other authors report visible tissue reactions involving immune infiltrates and fibrous capsular formation *in vivo* (Risbud et al., 2003). Another issue is that in contrast to alginate-based membranes, cellulose molecules can arrange closely together and form rigid structures which impact the permeability of the membranes. It has been shown that cellulose membranes prevent contact between activated complement proteins and the encapsulated islets (Risbud and Bhonde, 2001), but the low-permeability also delays insulin responses (Risbud et al., 2003).

Collagen is also able to form microcapsules for cell encapsulation. An advantage is that collagens are associated with minimal host responses (Yin et al., 2003). Although there are five major types of collagens, collagen type I is the most commonly applied polymer and also the most abundant type in the human body (Ramachandran, 1963; Lee et al., 2001). However, application of collagen in capsule manufacturing was limited by short-term mechanical stability and unstable permeability due to rapid enzymatic degradation post-transplantation (Szymanska and Winnicka, 2015). An enzyme resistant outer shell is required to maintain the integrity of the inner collagen core. A tetrapolymer of 2-hydroxyethyl methylacrylate—methacrylic acid—methyl methacrylate (HEMA– MAA–MMA) has been tested for this purpose (Chia et al., 2002; Yin et al., 2003). The capsules showed enhanced mechanical stability, a smoother surface and absence of protruding cells resulting in enhanced cell survival and function (Lahooti and Sefton, 2000; Chia et al., 2002). Other approaches involve application of crosslinkers to achieve long-term stability (Jorge-Herrero et al., 1999). Glutaradehyde was used as crosslinker to increase collagen stability but experiments were limited to *in vitro* studies due to severe host immune reactions (Marinucci et al., 2003). Success has been shown in reversing hyperglycemia in a diabetic rat model with hyaluronic acid-collagen hydrogel (HA-COL) encapsulated rat islets. These collagen based capsules were functional for up to 80 weeks with minimal fibrotic overgrowth or cellular rejection (Harrington et al., 2017). This might be due to more durable covalent crosslinks between HA and COL.

### Synthetic Polymers

Compared with natural polymers, synthetic materials do not suffer from batch-to-batch variations and can be chemically modified to achieve different physical, chemical and biological properties (Pişkin, 1995). However, toxic conditions such as non-physiological pH or temperature, UV illumination or harsh solvents needed during manufacturing of immunoisolating devices might compromise cell viability and function of cells in synthetic polymer-based capsules (Young et al., 2012; Headen et al., 2014; Esfahani et al., 2017). This is the reason why in the majority of studies with synthetic molecules focus on macrocapsules which can be manufactured in absence of islets. With macrocapsules in contrast to microcapsules membranes are first produced and islets loaded later when all solvents are washed out. This is more difficult with microcapsules were islets have to be packed in the capsules and polymerization has to occur when islets are embedded in the polymers.

Poly (ethylene glycol) (PEG) is one of the most versatile synthetic polymer and also the most commonly applied synthetic molecule for encapsulation of pancreatic islets (Hill et al., 1997; Cruise et al., 1999) and coating microcapsules (Villa et al., 2017). PEG is a water-soluble polymer, which allows application in microencapsulation in absence of too harsh solvents. Several groups have shown success with PEG as an immunoprotective membrane to prolong islet functional survival (Weber et al., 2009; Knobeloch et al., 2017). In contrast to most synthetic polymers, PEG forms hydrogels with a high water content that offers a mild microenvironment (Lutolf and Hubbell, 2005; Nuttelman et al., 2008) for encapsulated cells inside and a protein-resistant surface outside (Andrade and Hlady, 1987). Although without harsh solvents, a threat to islet survival still exists during the photopolymerization crosslinking process (Nguyen and West, 2002; Lin et al., 2009), which is associated with free radical generation and, consequently, functional cell loss (Sabnis et al., 2009). However, novel approaches have emerged. A microfluidic strategy for generation of PEG-maleimide (PEG-4MAL) was developed (Phelps et al., 2013). PEG-4MAL showed minimal toxicity to islets and inflammation *in vivo*. The PEG-4MAL microcapsule was generated by enveloping cells in the core of the PEG-4MAL solution and subsequently rapid crosslinking the droplets with dithiothreitol, which was associated with short residence time, minimal cell stress in absence of generation of free radicals. The system is still versatile as the network structure of PEG-4MAL can be tuned by applying PEG of different molecular weights to fine-tune molecular weight cut-off (Headen et al., 2014) Recently, an innovative four-arm PEG-4MAL polymer carrying vascular endothelial growth factor (VEGF) has been introduced for coating macrocapsules in order to accelerate device vascularization post-transplantation (Weaver et al., 2018).

Aliphatic polyesters have also been proposed for cell encapsulation (Cameron and Shaver, 2011) but its mechanical instability and difficult to tune permeability due to its biodegradability (Buchholz et al., 2016) has limited its application. Poly (lactic-co-glycolic acid) (PLGA) is a linear, polymerized aliphatic polyester that may overcome some issues as it possesses better biostability (Angelova and Hunkeler, 1999). However, PLGA still undergoes hydrolysis under physiological conditions and produces lactic acid and glycolic acid (Ding and Schwendeman, 2008) but these two monomers are non-toxic at normal physiological dose. It has been reported however that the degradation of PLGA lowered the surrounding pH and subsequently created an autocatalytic environment for proteins (van de Weert et al., 2000). The low pH in the microenvironment may influence the release of insulin and may even evoke host responses (Jiskoot et al., 2009). PLGA microencapsulated porcine islets have been xenotransplanted into diabetic rats and reduced hyperglycemia significantly, but hyperglycemia could be completely reversed (Abalovich et al., 2001). The PLGA encapsulated islets release less insulin than islets placed in diffusion chambers *in vitro*, which might illustrate a negative impact of PLGA degradation products on islet function or insulin releasing capacity (Abalovich et al., 2001). If the degradation of PLGA can be inhibited by modifying its structure, or its degree of crystallinity or amount of residual monomer (Xu et al., 2017) it still is a promising material for cell encapsulation because of its biocompatibility.

Another synthetic polymer that has been tested for cell-encapsulation is polyacrylate. This has been applied for both microencapsulation and macroencapsulation of pancreatic islets (Ronel et al., 1983; Sugamori and Sefton, 1989). Initial formulations of polyacrylate-based capsules had insufficient membrane permeability for water-soluble nutrients (Lahooti and Sefton, 1999). A modification that enhanced its applicability in cell encapsulation was that polyacrylate can be copolymerized with different acrylate units to tailor capsules with optimal biocompatibility and permeability (Stevenson and Sefton, 1987). To get an optimal rigidity and permeability, the hydrogel poly (2-hydroxyethyl methylacrylate) (HEMA) was copolymerized with the glassy poly (methyl methacrylate) (MMA) to manufacture the copolymer HEMA-MMA that can form flexible hydrogels for microcapsule generation (Babensee et al., 1992). A comparison of permeability between EUDRAGIT^®^ RL (a commercially available copolymer of ethyl acrylate, methyl methacrylate, and methacrylic acid ester) and HEMA-MMA indicated sufficient permeability offered by both of the two materials to insulin and glucose (Douglas and Sefton, 1990). However, it was too porous to protect enveloped cells for immunity and consequently only postponed graft destruction (Surzyn et al., 2009). The molecular weight cut-off of HEMA-MMA is around 100 kDa (Crooks et al., 1990), which cannot protect for escape of antigens and subsequent T cell activation (Surzyn et al., 2009). The application of HEMA-MMA microcapsules needs a novel approach to reduce and fine-tune permeability.

As a derivative of polyacrylate, polyacrylonitrile (PAN) was copolymerized with methallylsulfonate to produce AN69 (polyacrylonitrile-sodium methallylsulfonate) (Honiger et al., 1994). AN69 has been applied in macrocapsules (Kessler et al., 1991, 1992; Honiger et al., 1994; Colton, 1996). The AN69 membrane possesses optimal immunoisolation ability and is permeable to small molecular water-soluble substances (Sevastianov et al., 1984). However, the *in vivo* studies of AN69-based macrocapsules showed a reduced permeability for nutrients and insulin (Kessler et al., 1992), as a consequence of extreme protein adsorption (Silva et al., 2006).

Current challenges in application of many synthetic polymers for cell encapsulation are overcoming the use of hazardous solvents (Olabisi, 2015), reducing strong host responses (i.e., polyurethane and polypropylene) (George et al., 2002; Kawiak et al., 2003), or preventing fibrotic overgrowth (i.e., polyvinyl alcohol and polypropylene). Probably because of these issues combinations of natural and synthetic materials have attracted much attention from researchers. Several new concepts and multilayer encapsulation systems have emerged, which are discussed in following sections. However, first a common issue in application of synthetic and natural polymers needs to be discussed which is possible contaminations with endotoxins or better, pathogen-associated molecular patterns needs to be discussed.

### Pathogen-Associated Molecular Patterns (PAMPs) in Polymers

**Figure 3:**
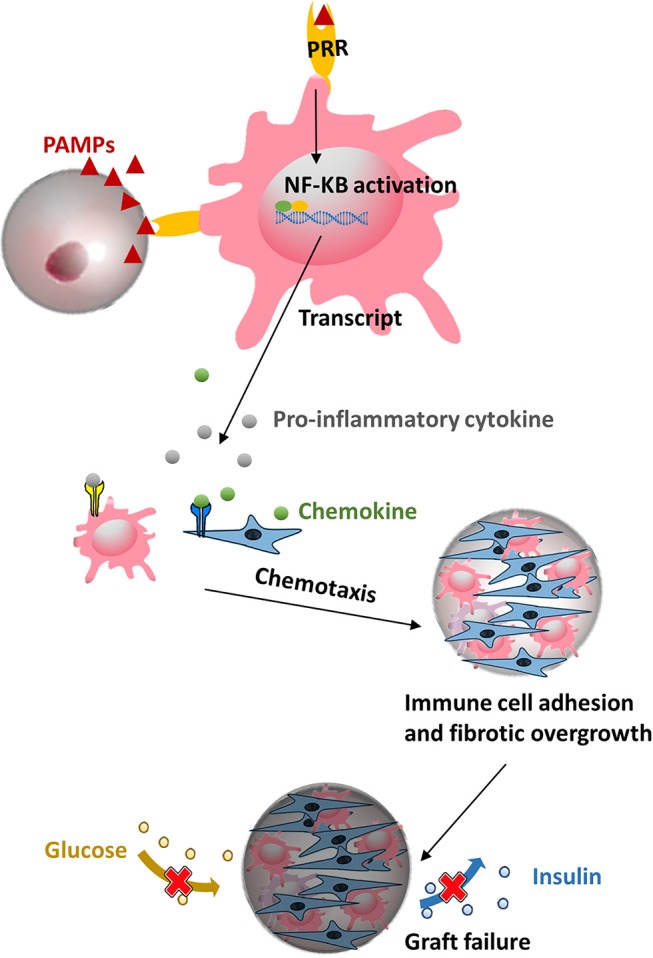
Microcapsule made from polymers might contain pathogen associated molecular patterns (PAMPs) that can be recognized by pattern-recognition receptors (PRRs) on macrophage and evoke subsequent a cascade of proinflammatory responses, ultimately leading to a pericapsular fibrotic overgrowth of capsules and necrosis of the islets.

A still ongoing and pertinent consideration in application of any polymer in cell-encapsulation is the need to use the polymers as pure as possible. Taking the most widely used natural polymer alginate as an example, all commercially available crude alginate contain proinflammatory PAMPs, including flagellin, lipopolysaccharide, peptidoglycan, lipoteichoic acid, and polyphenols (Paredes-Juárez et al., 2014b). Also other sources such as synthetic molecules i.e., polyethylene glycol was found in our assays to contain PAMPs. All of the above mentioned contaminants will play a negative role in host responses against capsules (Krishnan et al., 2017). During recent years it has been shown that these PAMPs (Paredes-Juarez et al., 2013, 2014a; Paredes-Juárez et al., 2014b) induce inflammatory responses in recipients either by diffusing out of the capsules or by being present at the capsule surface. This happens primarily via pattern-recognition receptors (PRRs) (Figure 3). After activation of PRRs on immune cells a cascade of intracellular signaling pathways are activated, leading to translocation of nuclear factor kappa-light-chain-enhancer of activated B cells (NF-κB) inducing inflammatory cytokine secretion, ultimately resulting in overgrowth of the capsules by immune-cells and fibroblasts (Kendall et al., 2004; Tam et al., 2006; Ménard et al., 2010; Paredes-Juárez et al., 2014b). Because fibrosis of the surface obstructs the ingress of nutrient and egress of waste, effective regulation of hyperglycemia is restricted to a limited period (de Vos et al., 1994, 2002b, 2012). Notably, apart from contaminants, it has been reported that uncrosslinked mannuronic acid polymers can trigger immune activation (Flo et al., 2002). For all these reasons, it is mandatory to apply purification procedures and quality assessment systems for purity of alginate (Paredes-Juarez et al., 2014a; de Vos, 2017; Orive et al., 2018).

There are a number of purification strategies published that obtain relatively endotoxin-free alginate. There are three mainstream classic “in-house” purification approaches (Klöck et al., 1994; De Vos et al., 1997b; Prokop and Wang, 1997). The protocol of de Vos starts with protein extraction with chloroform/butanol mixtures under acidic and neutral pH conditions (De Vos et al., 1997b). Prokop purified alginate by charcoal treatment and dialysis (Prokop and Wang, 1997), whereas the processes of forming, washing and dissolving alginate Ba^2+^ beads are applied in Klöck's protocol (Klöck et al., 1994). Purification procedures can reduce endotoxin, polyphenols, and proteins, but the final product differs greatly in degree of purity (Dusseault et al., 2006). In 2016 a novel purification strategy was added to the list of methods. This method is based on activated charcoal treatment, hydrophobic membrane filtration and dialysis (Sondermeijer et al., 2016). Using this approach, purified alginate was created that induced minimal foreign body reactions up to 1 month after implantation. In addition to purification a fast and efficient platform is needed to test the efficacy of purification. Paredes-Juarez et al. have published a platform that allows for identification of PRR activating capacity of polymers and finally identification of the type of contaminant in the polymers (Paredes-Juarez et al., 2014a). This eventually can lead to strategies to remove the contaminants. Despite the availability of several methods to purify alginates and to identify contaminants in polymers, it is still rarely used. This is however highly recommended as there are several lines of evidence that even polymers sold as ultrapure (Paredes-Juarez et al., 2013; Paredes-Juárez et al., 2014b) still contain endotoxins that might be responsible for inflammatory responses after implantation.

## Polymeric Engineering Approaches to Reduce Tissue Responses

### Multilayer Capsules

Due to shortcoming of some of the above discussed available polymers, the majority of researchers choose to produce microcapsule with application of combinations of molecules. Often these are applied in layer-by-layer systems (Tun et al., 1996; Schneider et al., 2001; Chia et al., 2002; Park et al., 2017). Alginate, as the most commonly used encapsulation materials, was in some confirmations, too porous to prevent penetration of IgG (Dembczynski and Jankowski, 2001) and some formulations were associated with low mechanical stability and higher surface roughness caused by cell protrusions after long term culture. Cationic polymers from chemical synthesis procedures were used to coat alginate-based capsules and overcome these issues. Commonly used examples are alginate coated with poly-l-lysine (PLL) (de Vos et al., 2002b), poly-L-Ornithine (Darrabie et al., 2005), PEG (Park et al., 2017), chitosan or agarose.

PLL was originally applied to decrease the pore size of alginate membranes and to enhance mechanical stability (De Vos et al., 1997a; Kendall and Opara, 2017). For many years, application of PLL was reported to be associated with enhanced immune responses against capsules. However, systemic studies with application of, for the field new, physics and chemical technologies such as Fourier-transform infrared spectroscopy (FT-IR), X-Ray Photoelectron Spectroscopy (XPS), and Time-of-Flight Secondary Ion Mass Spectrometry (ToF-SIMS) has revealed that PLL should be forced in a specific conformation to avoid responses. Any PLL that is not in the structure will bind cells in the vicinity of the capsules and provoke tissue responses. The following steps are essential to generate capsules with PLL that do not provoke responses. First, after gelification in a calcium solution alginate-based capsules have to be suspended in a low calcium high sodium buffer. During this step calcium on the surface of capsules is displaced by sodium that has lower affinity for alginate than PLL. This has to happen in the first few microns of the surface. Sodium will subsequently be substituted by PLL in a PLL-solution that lacks divalent cations. This process is temperature sensitive and should always be done in a consistent way. If done correctly, it creates a calcium alginate system that is composed of two layers, namely an alginate core and a layer of PLL-alginate complexes. There are three different binding modes in the outer layer, including (i) random coil formation between alginate and PLL, (ii) α-helicoidal structure between amide groups of PLL, and (iii) antiparallel β-sheet structure between amide groups of PLL (de Vos et al., 2002a; van Hoogmoed et al., 2003; Paredes-Juárez et al., 2014b). All PLL should be in this network which can be documented by FT-IR. By a stepwise approach and repeated implantations in mice it has been demonstrated that optimal biocompatible alginate-PLL capsules can be created as long as the PLL is in these confirmations (Juste et al., 2005). The PLL also improved the mechanical stability and permeability of alginate-based capsules (van Hoogmoed et al., 2003; Bhujbal et al., 2014b).

### Conformal Coating

As outlines in section Islets Encapsulation Technology many groups prefer to encapsulate islets in the smallest capsule possible to guarantee optimal nutritional supply to the enveloped islets (Orive et al., 2006; de Vos et al., 2009; Hall et al., 2011; Paredes-Juarez et al., 2013; Villa et al., 2017; Buchwald et al., 2018a). A recent study even suggest that the distance between islet-and surrounding fluid should be below 100 μm to allow optimal supply of nutrients (Iwata et al., 2018). These type of distances can be achieved with a technology called conformal coating (Tomei et al., 2014; Manzoli et al., 2017, 2018; Buchwald et al., 2018a). In addition to improving oxygen and nutrient transport conformal coating strategies also reduce the total transplant volume allowing implantation in other sites than the traditionally applied peritoneal cavity (Tomei et al., 2014; Buchwald et al., 2018b; Ernst et al., 2019). As this review does focus on polymers and tissue responses, we will discuss this subject in view of polymers applied and not current developments with this technology. Islet conformal coating approaches typically apply polyelectrolytes or complementary materials which are coated on a surface of cells or cell aggregates via intermolecular forces, i.e., electrostatic forces, hydrogen-bonds, or covalent linkages (Borges and Mano, 2014; Yamamoto et al., 2016). PEG was one of the first and still commonly applied polymers in islet conformal coating technologies. PEG is used in conformal coating techniques with photopolymerization (Cruise et al., 1999) microfluidic approaches (Tomei et al., 2014), via ester-bonding (Lazarjani et al., 2010), and via hydrogen-bonds (Wilson et al., 2010). In order to regulate permeability, multiple-arm PEG was developed. Islets conformally coated with this technique successfully corrected hyperglycemia for more than 100 days in mice (Rengifo et al., 2014; Giraldo et al., 2017). More recently, a heparin functionalized, 8-arm PEG was synthesized to coat islets with nanoscale barriers. This enhanced survival as it inhibited islet-cell apoptosis and promoted neovascularization *in vitro* (Lou et al., 2017). However, the potential anti-inflammatory effects of incorporated heparin, which is a well-known effect of heparin (Mao et al., 2017), was not discussed in this study.

During recent years the lay-by-layer (LBL) assembly with PEG has emerged as another promising alternative strategy (Ryan et al., 2017) to conformally coat islets. Theoretically this should overcome some limitation of the single-layer-PEG approach and in particular the potential harmful effects of PEG conformal coating techniques (Miura et al., 2006; Wilson et al., 2010; Chen et al., 2011) on mechanical instability (Itagaki et al., 2015; Yamamoto et al., 2016), and on sometimes inadequate immune-protection (Teramura et al., 2007). Polyelectrolytes applied in LBL coating can both be synthetic and natural polyelectrolytes (Granicka, 2014). In a recent study, acrylate modified cholesterol bearing pullulan (CHOPA) was employed to create a multilayer coating on β cell aggregates under mild polymerization conditions (Bal et al., 2018). In these CHOPA nanogels, pullulan can form immunologically inert gels without the use of toxic cations or other chemicals. In this system cholesterol units provide hydrophobic crosslinking points that promote self-assembly of polymeric particles (Bal et al., 2018). To reach an optimal equilibrium point of diffusion and immunoisolation, oppositely charged polymers (positively charged chitosan and negatively charged PSS) was applied in 9 layers on human islets (Syed et al., 2018). This system could induce normoglycemia for up to 180 days in a model of human to mice xenotransplantation with minimal immunocyte infiltration on the capsules (Syed et al., 2018). Also linear or star-shaped PEG derivatives are intensively studied for application in layer-by-layer approaches (Ryan et al., 2017; Perez-Basterrechea et al., 2018). Haque et al has built an coating layer with thiol-6-arm-PEG-lipid (SH-6-arm-PEG-lipid) and with gelatin-catechol to provide islets with a substitute for the extracellular matrix of islets and added three other coatings with 6-arm-PEG-SH, 6-arm-PEG-catechol, and linear PEG-SH respectively to provide immunoprotection (Haque et al., 2016). The multi-layer system preserved islet cell viability but the polymers showed minimal adsorption of human serum albumin, fibronectin, and immunoglobulin G. The system induced prolonged graft survival in a xenogeneic porcine-to-mouse model, which was further enhanced by applying an immunosuppressive cocktail (Haque et al., 2016). There is even efficacy shown in a xenogeneic monkey-to-mouse model in which 100% of the grafts survived for more than 150 days. After this period minimal or no immunocyte infiltration was observed (Haque et al., 2017). Given the potential severe side effects of generalized immunosuppression, a more recent study developed a controlled immunosuppressant FK506 release nanoparticle system using 3,4–dihydroxyphenethylamine (DOPA) conjugated PLGA–PEG to coat islet surfaces and to provide local immunosuppression (Pham et al., 2018). This study illustrates the potential of using layer-by-layer assembly as both barrier and carrier system for graft-survival promoting molecules.

### Anti-biofouling

In the post-transplantation period the host response starts with nonspecific protein adsorption and subsequent adhesion of immune cells and fibroblasts onto the capsule surface, a process termed “biofouling” (Harding and Reynolds, 2014). Several approaches have been explored to inhibit this issue with an approach called anti-biofouling which involves application of molecules on the surface of capsules to reduce protein adsorption. Most-studied strategies are based on application of low-biofouling polymers. Coated with hydrophilic polymeric materials the capsule surface is covered by a layer of water molecules, providing a highly resistant surface to protein adsorption (Kingshott and Griesser, 1999).

One of the most commonly applied molecules for anti-biofouling is PEG. PEG matrices can induce low protein adsorption but efficacy depends on chain density, length, and conformation (Michel et al., 2005; Unsworth et al., 2008). The protein resistance of a PEG surface proportionally increases with higher polymerization degrees and denser brush bristles on the surface (Andrade and Hlady, 1987; Cruje and Chithrani, 2014). PEG has been applied to coat alginate capsules to lower permeability and enhance mechanical stability but also served as anti-biofouling layer (Chen et al., 1998; Park et al., 2017). To coat alginate-based microcapsules, the PEG backbone was charged with added amine groups (NHs^+^), which can interact with naturally negatively charged alginate (Chen et al., 1998). In this way, PEG-amines can stably crosslink with alginate as a coating layer (Chen et al., 1998). Another group of investigators used mild glutaraldehyde (GA) treatment which increased the capsule strength, flexibility, and biocompatibility (Chandy et al., 1999). PEG coating brought many beneficial properties for cell encapsulation, including prevention of fibrotic overgrowth on the capsule surface (Chen et al., 1998). However, still tissue responses may occur which was further reduced by introducing immunosuppressive agents. In one of these approaches, rapamycin-PEG-coated alginate microcapsules inhibited non-specific binding and proliferation of macrophages *in vitro* and decreased fibrosis of capsules with more than 50% in a xenogeneic islet transplantation model (Park et al., 2017). Another approach using the protein-resistant property of PEG was by application of copolymers with PEG. Poly(ethylene glycol)-block-poly(l-lysine hydrochloride) (PEG-b-PLL) was coated on top of a proinflammatory, but immunoisolating, perm-selective alginate-PLL membrane (Spasojevic et al., 2014b). The diblock copolymer masked proinflammatory PLL and built an anti-fouling outer layer and successfully ameliorate host responses. A more recent study present a novel macroencapsulation strategy (Marchioli et al., 2017) that possibly induces anti-biofouling but also supports neovascularization while minimizing fibroblast adhesion. The technology applies two layers made of an anti-biofouling polyethyleneglycole diacrylate (PEGDA), and two pro-angiogenic growth factors conjugated to PEGDA. These two layers were covalently crosslinked and induced controlled release of basic fibroblast growth factor (bFGF) and vascular endothelial growth factor (VEGF) for up to 14 days (Marchioli et al., 2017) stimulating neovascularization.

### Polymer Brushes

**Figure 4:**
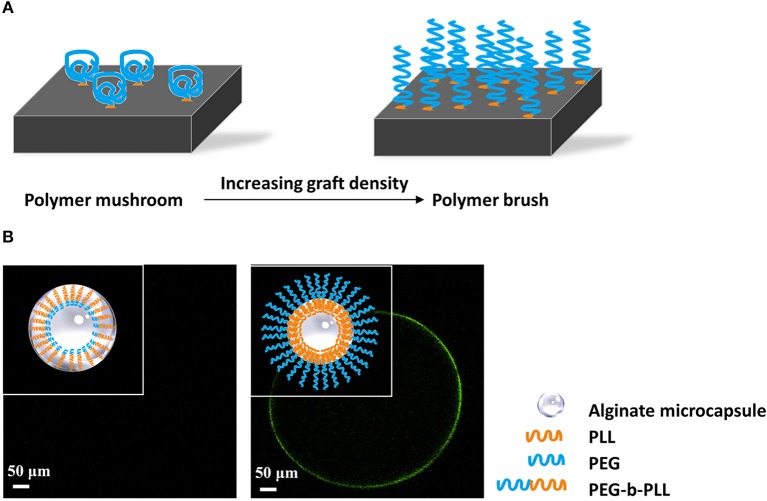
**(A)** Principle of formation of polymer brushes. At low grafting density polymers will have a mushroom conformation at the surface of capsules. When the grafting density increases and space becomes limited, the polymers will stretch and form a polymer brush that does not allow for protein and cell adhesion. **(B)** Schematic illustration of antibiofouling polymer brush surface formatted from PEG-b-PLL. PEG has to be long to prevent penetration into the alginate network and to stimulate stretching of the molecules on the surface (Spasojevic et al., 2014b). The outer PEG layer blocks shed unbound cytotoxic PLL and simultaneously provides a protein resistant surface, which showed antibiofouling properties *in vivo* studies.

An emerging new approach to reduce protein adsorption and cell-adhesion is application of polymer brushes. Polymer brushes consist of polymer chains that are densely tethered with other polymer chains on a surface (Figure 4A) (Feng and Huang, 2018). Polymer brushes form an ultrathin, solid coating (Kim and Jung, 2016). The polymer brush coating not only significantly changes the surface properties but also gives the surface new functionalities (Barbey et al., 2009). Spasojevic and colleagues showed a novel strategy combining the benefits of PLL and PEG by creating diblock co-polymers of poly(ethylene glycol)-block-poly(l-lysine hydrochloride) (PEG_454_-b-PLL_100_) (Spasojevic et al., 2014a). The copolymers bind with alginate with its positive charged PLL tail. PEG has to be long to prevent penetration into the alginate network and to stimulate stretching of the molecules on the surface (Figure 4B). The outer PEG layer blocks shed unbound cytotoxic PLL and simultaneously provides a biocompatible surface. Subsequent *in vivo* study proved the microcapsules have better biocompatibility illustrated by an absence of cell adherence (Spasojevic et al., 2014b).

Also other polymer brushes have been investigated recently. One of the studies coated soft chitosan surfaces with polymer brushes of oligo(ethylene glycol) methyl ether methacrylate and 2-hydroxyethyl methacrylate by photopolymerization (Buzzacchera et al., 2017). The novel polymer brush surface was reported to reduce protein adhesion and eliminated platelet activation and leukocyte adhesion (de los Santos Pereira et al., 2016; Buzzacchera et al., 2017). The application of diblock polymers is to our opinion a promising approach to combine advantages of different polymers but needs a multidisciplinary approach as in our hands uniform and complete coverage of the capsules surface with a brush was challenging.

## Accessory Cell Strategies to Reduce Tissue Responses

Often polymeric approaches are combined with pharmaceutical approaches to reduce tissue responses but during recent years a new emerging trend of applying and co-encapsulating “immunosuppressive” cells has shown some success. One of these immunosuppressive cell-types are T regulatory (Treg) cells. Tregs have been successfully immobilized on islet surfaces through streptavidin-biotin interactions (Gołab et al., 2014). This was done by first incubating the islets with a Biotin-PEG- succinimidyl valeric acid ester followed by an incubation with streptavidin. Subsequently, Treg cells were brought onto the islets (Gołab et al., 2014; Gliwinski et al., 2017). The islets coated with Treg-cells showed a lower glucose-stimulated insulin release than controls (Gołab et al., 2014). Although efficacy *in vivo* is not reported yet, this approach holds some promises as recruiting Treg cells by intramuscular co-transplantation of islets with a plasmid encoding Treg cell specific chemokine CCL22 was efficacious in preventing graft rejection (Vågesjö et al., 2015). Some success has also been shown in xenotransplantation with blockade of the costimulatory pathway of CD40/CD154, which inhibited T cell and B cell signaling (Ock et al., 2018; Do et al., 2019). In mice treated with this approach, increased numbers of Treg cells and an elevated anti-inflammatory cytokine profile was found around a porcine islet grafts (Wu et al., 2017). More recently, it was shown that Jagged-1, i.e., a potent immunomodulatory factor, immobilized on PEG-coated islet surfaces induced an increased population of Treg cells and a decreased level of proinflammatory cytokines *in vitro*, and an improved blood glucose control *in vivo* (Izadi et al., 2018). However, instead of enhancing the population of Treg cells, a recent study induced downregulation of proinflammatory T effector (Teff) cells by co-transplanting microgels conjugated with Fas-ligand on their surface (Headen et al., 2018). Fas, as a death receptor on the surface of T effector cells, can be activated by Fas-ligand resulting in an increased ratio of Treg to Teff (Headen et al., 2018). This system induced normoglycemia for more than 200 days in mice (Headen et al., 2018). The results show that a combination of polymeric encapsulation with recruitment of immune regulating cells might provide improved islet survival.

In addition to application of T-cells to regulate tissue responses, various endothelial cell types have been applied for co-encapsulation to promote survival of encapsulated cells (Ernst et al., 2019). Endothelial cells might have some benefits for islets as they have been shown to resist and neutralize reactive oxygen species, inhibit thrombogenesis, promote revascularization, and form extracellular matrices (Staels et al., 2016; Karimian et al., 2017). Co-transplanting these cells with islets has successfully promoted graft revascularization and promoted survival in several micro-and macroencapsulation approaches (Gupta and Sefton, 2011; Buitinga et al., 2016; Li et al., 2017). One study reports successful and expedited islet cell engraftment by coating islets with vascular endothelial cells (Barba-Gutierrez et al., 2016). During the last decade, also the application and co-encapsulated mesenchymal stem cells (MSCs) has been intensively studied in islet transplantation (Ben Nasr et al., 2015; Hirabaru et al., 2015; Unsal et al., 2015; Yoshimatsu et al., 2015; Cao et al., 2016). MSCs theoretically support angiogenesis and produces immunomodulatory molecules (Kim et al., 2019; Laporte et al., 2019), when co-transplanted with islets. Indeed co-encapsulation of MSCs have been shown to increase neovascularization and reduce islet cell death in both micro- and macroencapsulation approaches (Vériter et al., 2014; Borg et al., 2016; Buitinga et al., 2016; Bal et al., 2017; Hamilton et al., 2017) and might hold promises for improving graft survival.

## Immunomodulatory Materials

During recent years novel biomaterials have been designed that eventually might serve as immunomodulating polymers to reduce or prevent host reactions to encapsulated cell systems. One such an approach is application of Staudinger ligation chemistry to link immunomodulatory proteins with PEG. Staudinger chemistry, based on the specific crosslinking reaction between azide- and phosphine-labeled molecules, was successfully applied for conjugating different polymers (Hall et al., 2011) or bio-functional molecules with encapsulation polymers (Chen et al., 2011). Specifically, an amide bond was generated from an azide on protein and a specifically functionalized phosphine on triphenylphosphine-PEG. By this approach, thrombomodulin (TM) was bound with PEG, subsequently being immobilized on islet surfaces through streptavidin-biotin interactions. TM catalyzes the generation of activated protein C (APC) (Esmon, 2004), which possesses potent anti-inflammatory activity by inhibiting proinflammatory cytokines production in macrophages (Grey et al., 1994; Esmon, 2004). Co-immobilized TM induced protein C activation, which was similar to the activated protein C level catalyzed by endogenous TM in mouse pancreatic islets indicated reduction of inflammatory processes (Wilson et al., 2010). Chen and colleagues reported a different method to co-immobilized urokinase (UK) and TM on islet surfaces by PEG-conjugated phospholipids (Chen et al., 2011). Maleimide–PEG–lipid-anchored to the lipid bilayer membrane through hydrophobic interactions. Thiol (SH) groups on the SH-UK and SH-TM replaced maleimide groups and conjugated at the end of PEG chains on the cell membrane (Chen et al., 2011). The surface of islets coated with these membranes increased APC generation and released functional UK and TM, which reduced the instant blood-mediated inflammatory reactions after implantation and prolonged graft survival (Korsgren et al., 2008). In another approach to immunomodulate, hemoglobin (Hb-C) was crosslinked with PEG to scavenge nitric oxide (NO) and limit NO's negative biological actions (Han et al., 2002; Chae et al., 2004). Because of constraints that not every immunomodulator can be conjugated with polymers, an alternative strategy involves simple mixing immunomodulatory substances with polymers. Rapamycin has been trapped into PEG microcapsules and successfully prevented foreign body responses against capsules containing porcine islets (Park et al., 2017).

**Figure 5:**
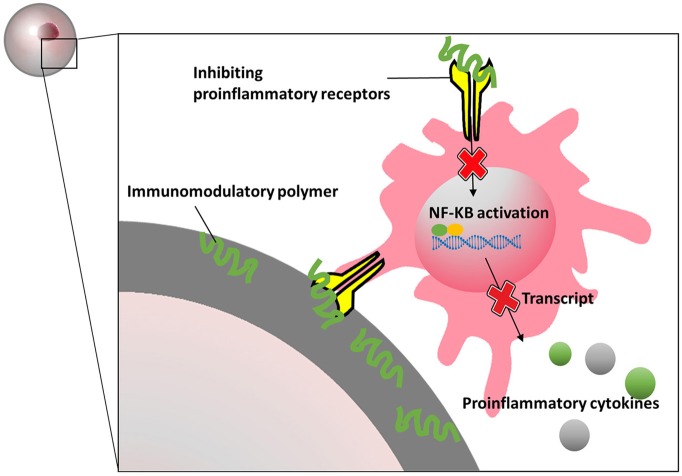
Schematics presentation of how immunomodulatory polymers block proinflammatory receptors and inhibit inflammatory signal pathways.

Also silk hydrogels have been shown to have immunomodulatory effects on macroencapsulated rat islets (Hamilton et al., 2017). Islets were seeded on silk scaffold and subsequently encapsulated in an alginate-Ba^2+^ network. An additional alginate-layer was added and cross-linked on the periphery of the scaffolds for immunoisolation (Kumar et al., 2017). The results indicate that blended silk hydrogel not only influenced islet viability, insulin secretion and endothelial cell maintenance, but also decreased production of proinflammatory cytokines *in vitro*. After injected with interleukin-4 (IL-4) and dexamethasone-loaded hydrogels, the silk macrocapsules showed a strong macrophage polarization toward a M2 phenotype which might provide an immunopermissive environment for the implants. A more recent study demonstrate that 2-aminoethyl methacrylate hydrochloride coupled to alginate can reduce tissue responses (Somo et al., 2018). By ionic crosslinking followed by exposure to ultraviolet light, 2-aminoethyl methacrylate hydrochloride modified alginate can be formed. The capsules were reportedly more mechanical stable than the alginate-beads and showed less inflammation on the surface of the beads after 3 weeks in LPS-stimulated rats (Somo et al., 2018). Meanwhile, in the field of intestinal immunity and bromatology, several heteropolysaccharides have been reported to possess immunomodulatory properties. Polysaccharide extracted from *Morinda citrifolia Linn* (Sousa et al., 2018), *Lentinula edodes* (Ren et al., 2018), *Schizophyllum commune* (Du et al., 2017), and lemon showed immunomodulatory effects. Most of these molecules bind to specific pro-inflammatory immune receptors which to our opinion might be a valuable approach to create immunomodulating capsules surfaces (Figure 5).

## Concluding Remarks and Future Perspectives

Although encapsulation in permselective membranes is a field that is around for more than three decades, important new polymeric approaches have emerged during recent years that create optimism that a technology can be developed that provokes minimal tissue responses and allows long term survival of encapsulated cells. The technology has revisited together with new approaches for creating a replenishable cell sources for curing endocrine diseases such as T1D. Some of these sources involve the use of xenogeneic tissue which might be particularly challenging in an encapsulation setting as indirect antigen presentation might be involved (Shin et al., 2014, 2015). Conceivable approaches to overcome influences of indirect antigen presentation might be application of the discussed polymer brushes and immunomodulating materials. With this approach either effector arms of the immune response can be blocked or adsorption of essential molecules to full-fill the response can be prevented. Also, lowering the permeability may be a suitable approach. The Beta-O2 device applied in pigs was having an Mw cutoff of about 80 kDa which might have been enough to prevent antigens responsible for indirect presentation to leak out (Neufeld et al., 2013). As such this review has attempted to demonstrate that rational choices for polymers and surface modification to modulate tissue responses and to prolong graft survival.

In addition to novel polymers to reduce tissue responses also other approaches have emerged. Some promising approaches are coating or co-encapsulation of nanoparticles for targeted and local drug delivery without systemical side-effects (Fang et al., 2014, 2017a, 2018; Dehaini et al., 2017; Zhang et al., 2017). Also approaches in which immune regulatory cells are applied in combination with encapsulation show promise although convincing *in vivo* results are not yet available. Apart from impact of polymers and tissue responses, long-term maintenance of islet cell viability is an important issue that requires much more attention by the scientific community. This is essential for graft function but also for reducing tissue responses as dead cells release danger-associated molecular patterns that provoke local tissue responses (Paredes-Juarez et al., 2015). A possible approach to prevent or reduce cell-death is by including extracellular matrix molecules (ECM) (Llacua et al., 2018b) in encapsulation systems (Del Guerra et al., 2001; Llacua et al., 2018a,c). ECM is damaged during islet isolation and has an enormous impact on survival of islets in encapsulated islet grafts (de Vos et al., 2016). ECM can stimulate cell proliferation, and eliminate known adverse factors.

Overall, current insight points to several potential successful strategies to reduce tissue responses against encapsulated islets grafts. This include novel or improved polymers but possibly also immune modulatory molecules or cells to allow long-term survival of encapsulated islet grafts. Although many long-term successes have been shown in several animal models there is consensus among insurance companies that 1-year survival is required with possibility to retrieve the graft before human application can be considered. To achieve this with in this review discussed approaches is to our opinion a realistic goal.

## Author Contributions

All authors listed have made a substantial, direct and intellectual contribution to the work, and approved it for publication.

### Conflict of Interest Statement

The authors declare that the research was conducted in the absence of any commercial or financial relationships that could be construed as a potential conflict of interest.

## Abbreviations

APC: activated protein C
BW: body weight
CHOPA: acrylate modified cholesterol bearing pullulan
DOPA, 3: 4–dihydroxyphenethylamine
FT-IR: fourier-transform infrared spectroscopy
G: α-L-guluronic acid
GA: glutaraldehyde
HA-COL: hyaluronic acid-collagen hydrogel
Hb-C: hemoglobin
HEMA: 2-hydroxyethyl methylacrylate
IgG: immunoglobulin G
M: β-D-mannuronic acid
MAA: methacrylic acid
MGC: methacrylated glycol chitosan
MMA: methyl methacrylate
MSCs: mesenchymal stem cells
NF-κB: nuclear factor kappa-light-chain-enhancer of activated B cells
NO: nitric oxide
PAMPs: pathogen-associated molecular patterns
PAN: polyacrylonitrile
PEG: poly (ethylene glycol)
PEG-4MAL: PEG-maleimide
PEG-b-PLL: poly(ethylene glycol)-block-poly(l-lysine hydrochloride
PEGDA: polyethyleneglycole diacrylate
PLGA: poly (lactic-co-glycolic acid)
PLL: poly-l-lysine
PRRs: pattern-recognition receptors
PSSa: polystyrene sulfonic acid PSSa
SH: thiol
T1D: type one diabetes mellitus
Teff: T effector
TM: thrombomodulin
TM: hrombomodulin
ToF-SIMS: Time-of-Flight Secondary Ion Mass Spectrometry
Treg: T regulatory
UK: urokinase
VEGF: vascular endothelial growth
XPS: X-Ray Photoelectron Spectroscopy.

## References

[B1] Abalovich A. Jatimliansky C. Diegex E. Arias M. Altamirano A. Amorena C.. (2001). Pancreatic islets microencapsulation with polylactide-co-glycolide. Transplant. Proc. 33, 1977–1979. 10.1016/S0041-1345(01)01918-2 11267595

[B2] Agudelo C. A. Teramura Y. Iwata H. (2009). Cryopreserved agarose-encapsulated islets as bioartificial pancreas: a feasibility study. Transplantation 87, 29–34. 10.1097/TP.0b013e318191b24b 19136888

[B3] American Diabetes Association (2018). Economic costs of diabetes in the U.S. in 2017. Diabetes Care 41, 917–928. 10.2337/dci18-0007 29567642 PMC5911784

[B4] Andrade J. D. Hlady V. (1987). Plasma protein adsorption: the big twelvea. Ann. N. Y. Acad. Sci. 516, 158–172. 10.1111/j.1749-6632.1987.tb33038.x 3439723

[B5] Angelova N. Hunkeler D. (1999). Rationalizing the design of polymeric biomaterials. Trends Biotechnol. 17, 409–421. 10.1016/S0167-7799(99)01356-6 10481173

[B6] Atkinson M. A. Eisenbarth G. S. Michels A. W. (2014). Type 1 diabetes. Lancet 383, 69–82. 10.1016/S0140-6736(13)60591-7 23890997 PMC4380133

[B7] Babensee J. E. De Boni U. Sefton M. V. (1992). Morphological assessment of hepatoma cells (HepG2) microencapsulated in a HEMA-MMA copolymer with and without Matrigel. J. Biomed. Mater. Res. 26, 1401–1418. 10.1002/jbm.820261102 1332972

[B8] Bal T. Nazli C. Okcu A. Duruksu G. Karaöz E. Kizilel S. (2017). Mesenchymal stem cells and ligand incorporation in biomimetic poly(ethylene glycol) hydrogels significantly improve insulin secretion from pancreatic islets. J. Tissue Eng. Regen. Med. 11, 694–703. 10.1002/term.1965 25393526

[B9] Bal T. Oran D. C. Sasaki Y. Akiyoshi K. Kizilel S. (2018). Sequential coating of insulin secreting beta cells within multilayers of polysaccharide nanogels. Macromol. Biosci. 18:e1800001. 10.1002/mabi.201800001 29575787

[B10] Barba-Gutierrez D. A. Daneri-Navarro A. Villagomez-Mendez J. J. Kanamune J. Robles-Murillo A. K. Sanchez-Enriquez S.. (2016). Facilitated engraftment of isolated islets coated with expanded vascular endothelial cells for islet transplantation. Transplant. Proc. 48, 669–672. 10.1016/j.transproceed.2016.02.036 27110026

[B11] Barbey R. Lavanant L. Paripovic D. Schüwer N. Sugnaux C. Tugulu S.. (2009). Polymer brushes via surface-initiated controlled radical polymerization: synthesis, characterization, properties, and applications. Chem. Rev. 109, 5437–5527. 10.1021/cr900045a 19845393

[B12] Barkai U. Rotem A. de Vos P. (2016). Survival of encapsulated islets: more than a membrane story. World J. Transplant. 6, 69–90. 10.5500/wjt.v6.i1.69 27011906 PMC4801806

[B13] Barkai U. Weir G. C. Colton C. K. Ludwig B. Bornstein S. R. Brendel M. D.. (2013). Enhanced oxygen supply improves islet viability in a new bioartificial pancreas. Cell Transplant. 22, 1463–1476. 10.3727/096368912X657341 23043896

[B14] Baruch L. Machluf M. (2006). Alginate–chitosan complex coacervation for cell encapsulation: Effect on mechanical properties and on long-term viability. Biopolymers 82, 570–579. 10.1002/bip.20509 16552738

[B15] Ben Nasr M. Vergani A. Avruch J. Liu L. Kefaloyianni E. D'Addio F.. (2015). Co-transplantation of autologous MSCs delays islet allograft rejection and generates a local immunoprivileged site. Acta Diabetol. 52, 917–927. 10.1007/s00592-015-0735-y 25808641 PMC4968999

[B16] Berney T. Ferrari-Lacraz S. Bühler L. Oberholzer J. Marangon N. Philippe J.. (2009). Long-term insulin-independence after allogeneic islet transplantation for type 1 diabetes: over the 10-year mark. Am. J. Transplant. 9, 419–423. 10.1111/j.1600-6143.2008.02481.x 19120085

[B17] Bhujbal S. V. de Haan B. Niclou S. P. de Vos P. (2014b). A novel multilayer immunoisolating encapsulation system overcoming protrusion of cells. Sci. Rep. 4:6856. 10.1038/srep06856 25358640 PMC4215319

[B18] Bhujbal S. V. Paredes-Juarez G. A. Niclou S. p. de Vos P. (2014a). Factors influencing the mechanical stability of alginate beads applicable for immunoisolation of mammalian cells. J. Mech. Behav. Biomed. Mater. 37, 196–208. 10.1016/j.jmbbm.2014.05.020 24951926

[B19] Bochenek M. A. Veiseh O. Vegas A. J. McGarrigle J. J. Qi M. Marchese E.. (2018). Alginate encapsulation as long-term immune protection of allogeneic pancreatic islet cells transplanted into the omental bursa of macaques. Nat. Biomed. Eng. 2, 810–821. 10.1038/s41551-018-0275-1 30873298 PMC6413527

[B20] Borg D. J. Welzel P. B. Grimmer M. Friedrichs J. Weigelt M. Wilhelm C.. (2016). Macroporous biohybrid cryogels for co-housing pancreatic islets with mesenchymal stromal cells. Acta Biomater. 44, 178–187. 10.1016/j.actbio.2016.08.007 27506126

[B21] Borges J. Mano J. F. (2014). Molecular interactions driving the layer-by-layer assembly of multilayers. Chem. Rev. 114, 8883–8942. 10.1021/cr400531v 25138984

[B22] Bragd J. Adamson U. Lins P. E. Wredling R. Oskarsson P. (2003). A repeated cross-sectional survey of severe hypoglycaemia in 178 Type 1 diabetes mellitus patients performed in 1984 and 1998. Diabet. Med. 20, 216–219. 10.1046/j.1464-5491.2003.00902.x 12675666

[B23] Bruni A. Gala-Lopez B. Pepper A. R. Abualhassan N. S. Shapiro A. J. (2014). Islet cell transplantation for the treatment of type 1 diabetes: recent advances and future challenges. Diabetes Metab. Syndr. Obes. 7, 211–223. 10.2147/DMSO.S50789 25018643 PMC4075233

[B24] Buchholz V. Agarwal S. Greiner A. (2016). Synthesis and enzymatic degradation of soft aliphatic polyesters. Macromol. Biosci. 16, 207–213. 10.1002/mabi.201500279 26401992

[B25] Buchwald P. Tamayo-Garcia A. Manzoli V. Tomei A. A. Stabler C. L. (2018a). Glucose-stimulated insulin release: parallel perifusion studies of free and hydrogel encapsulated human pancreatic islets. Biotechnol. Bioeng. 115, 232–245. 10.1002/bit.26442 28865118 PMC5699962

[B26] Buchwald P. Tomei A. A. Stabler C. L. (2018b). Predicting insulin secretion profiles for immunoisolating devices with transplanted islets. Diabetes 67(Suppl. 1):27-OR 10.2337/db18-27-OR

[B27] Buitinga M. Janeczek Portalska K. Cornelissen D. J. Plass J. Hanegraaf M. Carlotti F.. (2016). Coculturing human islets with proangiogenic support cells to improve islet revascularization at the subcutaneous transplantation site. Tissue Eng. Part A 22, 375–385. 10.1089/ten.tea.2015.0317 26871862 PMC4799698

[B28] Buzzacchera I. Vorobii M. Kostina N. Y. de los Santos Pereira A. Riedel T. Bruns M.. (2017). Polymer brush-functionalized chitosan hydrogels as antifouling implant coatings. Biomacromolecules 18, 1983–1992. 10.1021/acs.biomac.7b00516 28475307

[B29] Cameron D. J. Shaver M. P. (2011). Aliphatic polyester polymer stars: synthesis, properties and applications in biomedicine and nanotechnology. Chem. Soc. Rev. 40, 1761–1776. 10.1039/C0CS00091D 21082079

[B30] Cañibano-Hernández A. Saenz Del Burgo L. Espona-Noguera A. Orive G. Hernández R. M. Ciriza J.. (2019). Hyaluronic acid enhances cell survival of encapsulated insulin-producing cells in alginate-based microcapsules. Int. J. Pharm. 557, 192–198. 10.1016/j.ijpharm.2018.12.062 30597265

[B31] Cao X. K. Li R. Sun W. Ge Y. Liu B. L. (2016). Co-combination of islets with bone marrow mesenchymal stem cells promotes angiogenesis. Biomed. Pharmacother. 78, 156–164. 10.1016/j.biopha.2016.01.007 26898437

[B32] Chae S. Y. Lee M. Kim S. W. Bae Y. H. (2004). Protection of insulin secreting cells from nitric oxide induced cellular damage by crosslinked hemoglobin. Biomaterials 25, 843–850. 10.1016/S0142-9612(03)00605-7 14609673

[B33] Chandy T. Mooradian D. L. Rao G. H. (1999). Evaluation of modified alginate-chitosan-polyethylene glycol microcapsules for cell encapsulation. Artif. Organs 23, 894–903. 10.1046/j.1525-1594.1999.06244.x 10564287

[B34] Chen H. Teramura Y. Iwata H. (2011). Co-immobilization of urokinase and thrombomodulin on islet surfaces by poly(ethylene glycol)-conjugated phospholipid. J. Control. Release 150, 229–234. 10.1016/j.jconrel.2010.11.011 21108976

[B35] Chen J.-P. Chu I. M. Shiao M.-Y. Hsu B.R.-S. Fu S.-H. (1998). Microencapsulation of islets in PEG-amine modified alginate-poly(l-lysine)-alginate microcapsules for constructing bioartificial pancreas. J. Ferment. Bioeng. 86, 185–190. 10.1016/S0922-338X(98)80059-7

[B36] Chia S. M. Wan A. C. Quek C. H. Mao H. Q. Xu X. Shen L.. (2002). Multi-layered microcapsules for cell encapsulation. Biomaterials 23, 849–856. 10.1016/S0142-9612(01)00191-0 11774851

[B37] Cruje C. Chithrani D. B. (2014). Polyethylene glycol density and length affects nanoparticle uptake by cancer cells. J. Nanomed. Res. 1:00006 10.15406/jnmr.2014.01.00006

[B38] Choby B. (2017). Diabetes update: prevention and management of diabetes complications. FP Essent 456, 36–40. 28530383

[B39] Cieslinski D. A. David Humes H. (1994). Tissue engineering of a bioartificial kidney. Biotechnol. Bioeng. 43, 678–681. 10.1002/bit.260430718 18615768

[B40] Collaborative Islet Transplant Registry (2017). 10th Annual Report. Rockville, MD: The Emmes Corporation.

[B41] Colton C. K. (1996). Engineering challenges in cellencapsulation technology. Trends Biotechnol. 14, 158–162. 10.1016/0167-7799(96)10021-4 8645450

[B42] Cooper D. K. Matsumoto S. Abalovich A. Itoh T. Mourad N. I. Gianello P. R.. (2016). Progress in clinical encapsulated islet xenotransplantation. Transplantation 100, 2301–2308. 10.1097/TP.0000000000001371 27482959 PMC5077652

[B43] Crooks C. A. Douglas J. A. Broughton R. L. Sefton M. V. (1990). Microencapsulation of mammalian cells in a HEMA-MMA copolymer: effects on capsule morphology and permeability. J. Biomed. Mater. Res. 24, 1241–1262. 10.1002/jbm.820240908 2211747

[B44] Cruise G. M. Hegre O. D. Lamberti F. V. Hager S. R. Hill R. Scharp D. S.. (1999). *In vitro* and *in vivo* performance of porcine islets encapsulated in interfacially photopolymerized poly(ethylene glycol) diacrylate membranes. Cell Transplant. 8, 293–306. 10.1177/096368979900800310 10442742

[B45] Dantal J. Soulillou J. P. (2005). Immunosuppressive drugs and the risk of cancer after organ transplantation. N. Engl. J. Med. 352, 1371–1373. 10.1056/NEJMe058018 15800234

[B46] Darrabie M. D. Kendall W. F. Jr. Opara E. C. (2005). Characteristics of Poly-L-Ornithine-coated alginate microcapsules. Biomaterials 26, 6846–6852. 10.1016/j.biomaterials.2005.05.009 15955558

[B47] de los Santos Pereira A. Sheikh S. Blaszykowski C. Pop-Georgievski O. Fedorov K. Thompson M.. (2016). Antifouling polymer brushes displaying antithrombogenic surface properties. Biomacromolecules 17, 1179–1185. 10.1021/acs.biomac.6b00019 26882214

[B48] de Vos P. (2017). Historical perspectives and current challenges in cell microencapsulation. Methods Mol. Biol. 1479, 3–21. 10.1007/978-1-4939-6364-5_1 27738923

[B49] de Vos P. de Haan B. Pater J. Van Schilfgaarde R. (1996a). Association between capsule diameter, adequacy of encapsulation, and survival of microencapsulated rat islet allografts. Transplantation 62, 893–899. 10.1097/00007890-199610150-00004 8878380

[B50] De Vos P. De Haan B. Van Schilfgaarde R. (1997a). Effect of the alginate composition on the biocompatibility of alginate-polylysine microcapsules. Biomaterials 18, 273–278. 10.1016/S0142-9612(96)00135-4 9031730

[B51] De Vos P. De Haan B. Wolters G. H. Van Schilfgaarde R. (1996b). Factors influencing the adequacy of microencapsulation of rat pancreatic islets. Transplantation 62, 888–893. 10.1097/00007890-199610150-00003 8878379

[B52] de Vos P. de Haan B. J. de Haan A. van Zanten J. Faas M. M. (2004). Factors influencing functional survival of microencapsulated islet grafts. Cell Transplant. 13, 515–524. 10.3727/000000004783983738 15565864

[B53] De Vos P. De Haan B. J. Wolters G. H. Strubbe J. H. Van Schilfgaarde R. (1997b). Improved biocompatibility but limited graft survival after purification of alginate for microencapsulation of pancreatic islets. Diabetologia 40, 262–270. 10.1007/s001250050673 9084963

[B54] de Vos P. Hoogmoed C. G. Busscher H. J. (2002a). Chemistry and biocompatibility of alginate-PLL capsules for immunoprotection of mammalian cells. J. Biomed. Mater. Res. 60, 252–259. 10.1002/jbm.10060 11857431

[B55] de Vos P. Lazarjani H. A. Poncelet D. Faas M. M. (2014). Polymers in cell encapsulation from an enveloped cell perspective. Adv. Drug Deliv. Rev. 67–68, 15–34. 10.1016/j.addr.2013.11.005 24270009

[B56] de Vos P. Marchetti P. (2002). Encapsulation of pancreatic islets for transplantation in diabetes: the untouchable islets. Trends Mol. Med. 8, 363–366. 10.1016/S1471-4914(02)02381-X 12127717

[B57] de Vos P. Smink A. M. Paredes G. Lakey J. R. Kuipers J. Giepmans B. N.. (2016). Enzymes for pancreatic islet isolation impact chemokine-production and polarization of insulin-producing beta-cells with reduced functional survival of immunoisolated rat islet-allografts as a consequence. PLoS ONE 11:e0147992. 10.1371/journal.pone.0147992 26824526 PMC4732769

[B58] de Vos P. Spasojevic M. de Haan B. J. Faas M. M. (2012). The association between *in vivo* physicochemical changes and inflammatory responses against alginate based microcapsules. Biomaterials 33, 5552–5559. 10.1016/j.biomaterials.2012.04.039 22560199

[B59] de Vos P. Spasojevic M. Faas M. M. (2010). Treatment of diabetes with encapsulated islets. Adv. Exp. Med. Biol. 670, 38–53. 10.1007/978-1-4419-5786-3_5 20384217

[B60] de Vos P. van Hoogmoed C. G. de Haan B. J. Busscher H. J. (2002b). Tissue responses against immunoisolating alginate-PLL capsules in the immediate posttransplant period. J. Biomed. Mater. Res. 62, 430–437. 10.1002/jbm.10345 12209929

[B61] de Vos P. Wolters G. H. van Schilfgaarde R. (1994). Possible relationship between fibrotic overgrowth of alginate-polylysine-alginate microencapsulated pancreatic islets and the microcapsule integrity. Transplant. Proc. 26, 782–783. 7513474

[B62] de Vos P. Bucko M. Gemeiner P. Navrátil M. Svitel J. Faas M.. (2009). Multiscale requirements for bioencapsulation in medicine and biotechnology. Biomaterials 30, 2559–2570. 10.1016/j.biomaterials.2009.01.014 19201460

[B63] Dehaini D. Wei X. Fang R. H. Masson S. Angsantikul P. Luk B. T.. (2017). Erythrocyte–platelet hybrid membrane coating for enhanced nanoparticle functionalization. Adv. Mater. 29:1606209. 10.1002/adma.201606209 28199033 PMC5469720

[B64] Del Guerra S. Bracci C. Nilsson K. Belcourt A. Kessler L. Lupi R.. (2001). Entrapment of dispersed pancreatic islet cells in CultiSpher-S macroporous gelatin microcarriers: preparation, *in vitro* characterization, and microencapsulation. Biotechnol. Bioeng. 75, 741–744. 10.1002/bit.10053 11745153

[B65] Dembczynski R. Jankowski T. (2001). Determination of pore diameter and molecular weight cut-off of hydrogel-membrane liquid-core capsules for immunoisolation. J. Biomater. Sci. Polym. Ed. 12, 1051–1058. 10.1163/156856201753252552 11787521

[B66] Ding A. G. Schwendeman S. P. (2008). Acidic microclimate pH distribution in PLGA microspheres monitored by confocal laser scanning microscopy. Pharm. Res. 25, 2041–2052. 10.1007/s11095-008-9594-3 18622692 PMC4269251

[B67] Do P. Beckwith K. A. Cheney C. Tran M. Beaver L. Griffin B. G.. (2019). Leukemic B cell CTLA-4 suppresses costimulation of T cells. J. Immunol. 202, 2806–2816. 10.4049/jimmunol.1801359 30910862 PMC6478536

[B68] Douglas J. A. Sefton M. V. (1990). The permeability of EUDRAGIT RL and HEMA–MMA microcapsules to glucose and inulin. Biotechnol. Bioeng. 36, 653–664. 10.1002/bit.260360702 18597256

[B69] Du B. Yang Y. Bian Z. Xu B. (2017). Characterization and anti-inflammatory potential of an exopolysaccharide from submerged mycelial culture of schizophyllum commune. Front Pharmacol. 8, 252–252. 10.3389/fphar.2017.00252 28555107 PMC5430044

[B70] Dufrane D. Goebbels R. M. Saliez A. Guiot Y. Gianello P. (2006). Six-month survival of microencapsulated pig islets and alginate biocompatibility in primates: proof of concept. Transplantation 81, 1345–1353. 10.1097/01.tp.0000208610.75997.20 16699465

[B71] Dupuy B. Baquey A. Baquey C. Ducassou D. (1990). Lack of responsiveness to glucose of microencapsulated islets of Langerhans after three weeks' implantation in the rat—influence of the complement AU - Gin, H. J. Microencapsul. 7, 341–346. 10.3109/02652049009021844 2117059

[B72] Dusseault J. Tam S. K. Ménard M. Polizu S. Jourdan G. Yahia L.. (2006). Evaluation of alginate purification methods: effect on polyphenol, endotoxin, and protein contamination. J. Biomed. Mater. Res. A 76, 243–251. 10.1002/jbm.a.30541 16265647

[B73] Duvivier-Kali V. F. Omer A. Parent R. J. O'Neil J. J. Weir G. C. (2001). Complete protection of islets against allorejection and autoimmunity by a simple barium-alginate membrane. Diabetes 50, 1698–1705. 10.2337/diabetes.50.8.1698 11473027

[B74] Ekberg H. Bernasconi C. Tedesco-Silva H. Vitko S. Hugo C. Demirbas A.. (2009). Calcineurin inhibitor minimization in the Symphony study: observational results 3 years after transplantation. Am. J. Transplant. 9, 1876–1885. 10.1111/j.1600-6143.2009.02726.x 19563339

[B75] Ekser B. Cooper D. K. C. Tector A. J. (2015). The need for xenotransplantation as a source of organs and cells for clinical transplantation. Int. J. Surg. 23(Pt B), 199–204. 10.1016/j.ijsu.2015.06.066 26188183 PMC4684733

[B76] Ernst A. U. Bowers D. T. Wang L. H. Shariati K. Plesser M. D. Brown N. K.. (2019). Nanotechnology in cell replacement therapies for type 1 diabetes. Adv. Drug Deliv. Rev. 10.1016/j.addr.2019.01.013 30716349 PMC6677642

[B77] Esfahani R. R. Jun H. Rahmani S. Miller A. Lahann J. (2017). Microencapsulation of live cells in synthetic polymer capsules. ACS Omega 2, 2839–2847. 10.1021/acsomega.7b00570 30023677 PMC6044854

[B78] Esmon C. T. (2004). Crosstalk between inflammation and thrombosis. Maturitas 47, 305–314. 10.1016/j.maturitas.2003.10.015 15063484

[B79] Fang R. H. Hu C. M. Luk B. T. Gao W. Copp J. A. Tai Y.. (2014). Cancer cell membrane-coated nanoparticles for anticancer vaccination and drug delivery. Nano Lett. 14, 2181–2188. 10.1021/nl500618u 24673373 PMC3985711

[B80] Fang R. H. Jiang Y. Fang J. C. Zhang L. (2017a). Cell membrane-derived nanomaterials for biomedical applications. Biomaterials 128, 69–83. 10.1016/j.biomaterials.2017.02.041 28292726 PMC5417338

[B81] Fang R. H. Kroll A. V. Gao W. Zhang L. (2018). Cell membrane coating nanotechnology. Adv. Mater. 30:e1706759. 10.1002/adma.201706759 29582476 PMC5984176

[B82] Fang W. Bi D. Zheng R. Cai N. Xu H. Zhou R.. (2017b). Identification and activation of TLR4-mediated signalling pathways by alginate-derived guluronate oligosaccharide in RAW264.7 macrophages. Sci. Rep. 7, 1663–1663. 10.1038/s41598-017-01868-0 28490734 PMC5431981

[B83] Feng C. Huang X. (2018). Polymer brushes: efficient synthesis and applications. Acc. Chem. Res. 51, 2314–2323. 10.1021/acs.accounts.8b00307 30137964

[B84] Fernández-Cossío S. León-Mateos A. Sampedro F. G. Oreja M. T. (2007). Biocompatibility of agarose gel as a dermal filler: histologic evaluation of subcutaneous implants. Plast. Reconstr. Surg. 120, 1161–1169. 10.1097/01.prs.0000279475.99934.71 17898590

[B85] Flo T. H. Ryan L. Latz E. Takeuchi O. Monks B. G. Lien E.. (2002). Involvement of toll-like receptor (TLR) 2 and TLR4 in cell activation by mannuronic acid polymers. J. Biol. Chem. 277, 35489–35495. 10.1074/jbc.M201366200 12089142

[B86] Gazda L. S. Vinerean H. V. Laramore M. A. Hall R. D. Carraway J. W. Smith B. H. (2014). Pravastatin improves glucose regulation and biocompatibility of agarose encapsulated porcine islets following transplantation into pancreatectomized dogs. J. Diabetes Res. 2014:405362. 10.1155/2014/405362 24963494 PMC4055154

[B87] George S. Nair P. D. Risbud M. V. Bhonde R. R. (2002). Nonporous polyurethane membranes as islet immunoisolation matrices – biocompatibility studies. J. Biomater. Appl. 16, 327–340. 10.1106/088532802024249 12099511

[B88] Giraldo J. A. Molano R. D. Rengifo H. R. Fotino C. Gattás-Asfura K. M. Pileggi A.. (2017). The impact of cell surface PEGylation and short-course immunotherapy on islet graft survival in an allogeneic murine model. Acta Biomater. 49, 272–283. 10.1016/j.actbio.2016.11.060 27915019 PMC5253093

[B89] Gliwinski M. Iwaszkiewicz-Grześ D. Trzonkowski P. (2017). Cell-based therapies with T regulatory cells. BioDrugs Clin. Immunother. Biopharm. Gene Ther. 31, 335–347. 10.1007/s40259-017-0228-3 28540499 PMC5548816

[B90] Gmyr V. Bonner C. Moerman E. Tournoys A. Delalleau N. Quenon A.. (2017). Human recombinant antithrombin (ATryn((R))) administration improves survival and prevents intravascular coagulation after intraportal islet transplantation in a piglet model. Cell Transplant. 26, 309–317. 10.3727/096368916X693554 27938471 PMC5657771

[B91] Gołab K. Kizilel S. Bal T. Hara M. Zielinski M. Grose R.. (2014). Improved coating of pancreatic islets with regulatory T cells to create local immunosuppression by using the biotin-polyethylene glycol-succinimidyl valeric acid ester molecule. Transplant. Proc. 46, 1967–1971. 10.1016/j.transproceed.2014.05.075 25131084 PMC4435942

[B92] Grace K. Arjun N. Shu-Meng K. Rahul K. So-Ra L. Michael A. (2016). “Alginate composition, temperature, and presence of islet tissue influence microcapsule permeability,” in Frontiers in Bioengineering and Biotechnology 10th World Biomaterials Congress, Vol. 4 (Montréal, QC). 10.3389/conf.FBIOE.2016.01.03002

[B93] Granicka L. H. (2014). Nanoencapsulation of cells within multilayer shells for biomedical applications. J. Nanosci. Nanotechnol. 14, 705–716. 10.1166/jnn.2014.9106 24730291

[B94] Grey S. T. Tsuchida A. Hau H. Orthner C. L. Salem H. H. Hancock W. W. (1994). Selective inhibitory effects of the anticoagulant activated protein C on the responses of human mononuclear phagocytes to LPS, IFN-gamma, or phorbol ester. J. Immunol. 153, 3664–3672. 7523500

[B95] Gupta R. Sefton M. V. (2011). Application of an endothelialized modular construct for islet transplantation in syngeneic and allogeneic immunosuppressed rat models. Tissue Eng. Part A 17, 2005–2015. 10.1089/ten.tea.2010.0542 21449709 PMC3142635

[B96] Hall K. K. Gattás-Asfura K. M. Stabler C. L. (2011). Microencapsulation of islets within alginate/poly(ethylene glycol) gels cross-linked via Staudinger ligation. Acta Biomater. 7, 614–624. 10.1016/j.actbio.2010.07.016 20654745 PMC2974964

[B97] Hamilton D. C. Shih H. H. Schubert R. A. Michie S. A. Staats P. N. Kaplan D. L.. (2017). A silk-based encapsulation platform for pancreatic islet transplantation improves islet function *in vivo*. J. Tissue Eng. Regen. Med. 11, 887–895. 10.1002/term.1990 25619945

[B98] Han T. H. Hyduke D. R. Vaughn M. W. Fukuto J. M. Liao J. C. (2002). Nitric oxide reaction with red blood cells and hemoglobin under heterogeneous conditions. Proc. Natl. Acad. Sci. U.S.A. 99, 7763–7768. 10.1073/pnas.122118299 12032357 PMC124345

[B99] Haque M. R. Jeong J. H. Byun Y. (2016). Combination strategy of multi-layered surface camouflage using hyperbranched polyethylene glycol and immunosuppressive drugs for the prevention of immune reactions against transplanted porcine islets. Biomaterials 84, 144–156. 10.1016/j.biomaterials.2016.01.039 26828680

[B100] Haque M. R. Kim J. Park H. Lee H. S. Lee K. W. Al-Hilal T. A.. (2017). Xenotransplantation of layer-by-layer encapsulated non-human primate islets with a specified immunosuppressive drug protocol. J. Control. Release 258, 10–21. 10.1016/j.jconrel.2017.04.021 28433740

[B101] Harding J. L. Reynolds M. M. (2014). Combating medical device fouling. Trends Biotechnol. 32, 140–146. 10.1016/j.tibtech.2013.12.004 24438709

[B102] Harrington S. Williams J. Rawal S. Ramachandran K. Stehno-Bittel L. (2017). Hyaluronic acid/collagen hydrogel as an alternative to alginate for long-term immunoprotected islet transplantation. Tissue Eng. Part A 23, 1088–1099. 10.1089/ten.tea.2016.0477 28142500 PMC6112162

[B103] Headen D. M. Aubry G. Lu H. García A. J. (2014). Microfluidic-based generation of size-controlled, biofunctionalized synthetic polymer microgels for cell encapsulation. Adv. Mater. 26, 3003–3008. 10.1002/adma.201304880 24615922 PMC4058833

[B104] Headen D. M. Woodward K. B. Coronel M. M. Shrestha P. Weaver J. D. Zhao H.. (2018). Local immunomodulation with Fas ligand-engineered biomaterials achieves allogeneic islet graft acceptance. Nat. Mater. 17, 732–739. 10.1038/s41563-018-0099-0 29867165 PMC6060019

[B105] Heng W. S. Wan L. S. C. (1997). Effect of cellulose derivatives on alginate micro spheresprepared by emulsification AU - Chanp, L. W. J. Microencapsul. 14, 545–555. 10.3109/02652049709006808 9292431

[B106] Hill R. S. Cruise G. M. Hager S. R. Lamberti F. V. Yu X. Garufis C. L.. (1997). Immunoisolation of adult porcine islets for the treatment of diabetes mellitus. The use of photopolymerizable polyethylene glycol in the conformal coating of mass-isolated porcine islets. Ann. N. Y. Acad. Sci. 831, 332–343. 10.1111/j.1749-6632.1997.tb52208.x 9616725

[B107] Hillberg A. L. Kathirgamanathan K. Lam J. B. Law L. Y. Garkavenko O. Elliott R. B. (2013). Improving alginate-poly-L-ornithine-alginate capsule biocompatibility through genipin crosslinking. J. Biomed. Mater. Res. B Appl. Biomater. 101, 258–268. 10.1002/jbm.b.32835 23166035

[B108] Hillberg A. L. Oudshoorn M. Lam J. B. Kathirgamanathan K. (2015). Encapsulation of porcine pancreatic islets within an immunoprotective capsule comprising methacrylated glycol chitosan and alginate. J. Biomed. Mater. Res. Part B Appl. Biomater. 103, 503–518. 10.1002/jbm.b.33185 24915784

[B109] Hirabaru M. Kuroki T. Adachi T. Kitasato A. Ono S. Tanaka T.. (2015). A method for performing islet transplantation using tissue-engineered sheets of islets and mesenchymal stem cells. Tissue Eng. Part C Methods 21, 1205–1215. 10.1089/ten.tec.2015.0035 26066973 PMC4663636

[B110] Hirsch I. B. (2009). Realistic expectations and practical use of continuous glucose monitoring for the endocrinologist. J. Clin. Endocrinol. Metab. 94, 2232–2238. 10.1210/jc.2008-2625 19383778

[B111] Honiger J. Darquy S. Reach G. Muscat E. Thomas M. Collier C. (1994). Preliminary report on cell encapsulation in a hydrogel made of a biocompatible material, AN69, for the development of a bioartificial pancreas. Int. J. Artif. Organs 17, 46–52. 10.1177/039139889401700108 8188399

[B112] Itagaki T. Arima Y. Kuwabara R. Kitamura N. Iwata H. (2015). Interaction between cells and poly(ethylene glycol)-lipid conjugates. Colloids Surfaces B Biointerfaces 135, 765–773. 10.1016/j.colsurfb.2015.08.014 26342322

[B113] Iwata H. Arima Y. Tsutsui Y. (2018). Design of bioartificial pancreases from the standpoint of oxygen supply. Artif. Organs 42, E168–E185. 10.1111/aor.13106 29611212

[B114] Iwata H. Kobayashi K. Takagi T. Oka T. Yang H. Amemiya H.. (1994). Feasibility of agarose microbeads with xenogeneic islets as a bioartificial pancreas. J. Biomed. Mater. Res. 28, 1003–1011. 10.1002/jbm.820280905 7814428

[B115] Iwata H. Takagi T. Amemiya H. (1992a). Agarose microcapsule applied in islet xenografts (hamster to mouse). Transplant. Proc. 24, 952. 1604680

[B116] Iwata H. Takagi T. Amemiya H. Shimizu H. Yamashita K. Kobayashi K. (1992b). Agarose for a bioartificial pancreas. J. Biomed. Mater. Res. 26, 967–977. 10.1002/jbm.820260711 1607377

[B117] Izadi Z. Hajizadeh-Saffar E. Hadjati J. Habibi-Anbouhi M. Ghanian M. H. Sadeghi-Abandansari H.. (2018). Tolerance induction by surface immobilization of Jagged-1 for immunoprotection of pancreatic islets. Biomaterials 182, 191–201. 10.1016/j.biomaterials.2018.08.017 30134210

[B118] Jiskoot W. van Schie R. M. Carstens M. G. Schellekens H. (2009). Immunological risk of injectable drug delivery systems. Pharm. Res. 26, 1303–1314. 10.1007/s11095-009-9855-9 19247815

[B119] Jorge-Herrero E. Fernández P. Turnay J. Olmo N. Calero P. García R. (1999). Influence of different chemical cross-linking treatments on the properties of bovine pericardium and collagen. Biomaterials 20, 539–545. 10.1016/S0142-9612(98)90205-8 10213357

[B120] Juste S. Lessard M. Henley N. Ménard M. Hallé J. P. (2005). Effect of poly-L-lysine coating on macrophage activation by alginate-based microcapsules: assessment using a new *in vitro* method. J. Biomed. Mater. Res. A 72, 389–398. 10.1002/jbm.a.30254 15669081

[B121] Karimian M. S. Pirro M. Johnston T. P. Majeed M. Sahebkar A. (2017). Curcumin and endothelial function: evidence and mechanisms of protective effects. Curr. Pharm. Des. 23, 2462–2473. 10.2174/1381612823666170222122822 28228072

[B122] Kawiak J. Snochowski M. Wójcicki J. M. Sabalinska S. Werynski A. (2003). Polypropylene hollow fiber for cells isolation: methods for evaluation of diffusive transport and quality of cells encapsulation AU - Granicka, Ludomira, H. Arti. Cells Blood Substit. Biotechnol. 31, 249–262. 10.1081/BIO-120023156 12906307

[B123] Kendall W. F. Darrabie M. D. El-Shewy H. M. Opara E. C. (2004). Effect of alginate composition and purity on alginate microspheres. J. Microencapsul. 21, 821–828. 10.1080/02652040400015452 15799538

[B124] Kendall W. F. Opara E. C. (2017). Polymeric materials for perm-selective coating of alginate microbeads. Methods Mol. Biol. 1479, 95–109. 10.1007/978-1-4939-6364-5_7 27738929

[B125] Kessler L. Aprahamian M. Keipes M. Damgé C. Pinget M. Poinsot D. (1992). Diffusion properties of an artificial membrane used for Langerhans islets encapsulation: an *in vitro* test. Biomaterials 13, 44–49. 10.1016/0142-9612(92)90094-5 1543808

[B126] Kessler L. Pinget M. Aprahamian M. Dejardin P. Damgé C. (1991). *In vitro* and *in vivo* studies of the properties of an artificial membrane for pancreatic islet encapsulation. Horm. Metab. Res. 23, 312–317. 10.1055/s-2007-1003685 1774016

[B127] Kim J.-S. Jung Y. Kim S. H. Shin J.-S. Kim S. H. Park C.-G. (2019). Vascularization of PLGA-based bio-artificial beds by hypoxia-preconditioned mesenchymal stem cells for subcutaneous xenogeneic islet transplantation. Xenotransplantation 26:e12441. 10.1111/xen.12441 30054954

[B128] Kim W. Jung J. (2016). Polymer brush: a promising grafting approach to scaffolds for tissue engineering. BMB Rep. 49, 655–661. 10.5483/BMBRep.2016.49.12.166 27697112 PMC5346310

[B129] Kingshott P. Griesser H. J. (1999). Surfaces that resist bioadhesion. Curr. Opin. Solid State Mater. Sci. 4, 403–412. 10.1016/S1359-0286(99)00018-2

[B130] Klöck G. Frank H. Houben R. Zekorn T. Horcher A. Siebers U.. (1994). Production of purified alginates suitable for use in immunoisolated transplantation. Appl. Microbiol. Biotechnol. 40, 638–643. 10.1007/BF00173321 7764423

[B131] Knobeloch T. Abadi S. E. M. Bruns J. Zustiak S. P. Kwon G. (2017). Injectable polyethylene glycol hydrogel for islet encapsulation: an *in vitro* and *in vivo* characterization. Biomed. Phys. Eng. Express 3:035022. 10.1088/2057-1976/aa742b 29527325 PMC5842952

[B132] Kobayashi T. Aomatsu Y. Iwata H. Kin T. Kanehiro H. Hisanaga M.. (2003). Indefinite islet protection from autoimmune destruction in nonobese diabetic mice by agarose microencapsulation without immunosuppression1. Transplantation 75, 619–625. 10.1097/01.TP.0000053749.36365.7E 12640299

[B133] Komatsu H. Kandeel F. Mullen Y. (2018). Impact of oxygen on pancreatic islet survival. Pancreas 47, 533–543. 10.1097/MPA.0000000000001050 29621044 PMC5943071

[B134] Korsgren O. Lundgren T. Felldin M. Foss A. Isaksson B. Permert J.. (2008). Optimising islet engraftment is critical for successful clinical islet transplantation. Diabetologia 51, 227–232. 10.1007/s00125-007-0868-9 18040664

[B135] Krishnan R. Ko D. Foster C. E. III. Liu W. Smink A. M. de Haan B.. (2017). Immunological challenges facing translation of alginate encapsulated porcine islet xenotransplantation to human clinical trials. Methods Mol. Biol. 1479, 305–333. 10.1007/978-1-4939-6364-5_24 27738946

[B136] Kubota N. Tatsumoto N. Sano T. Toya K. (2000). A simple preparation of half N-acetylated chitosan highly soluble in water and aqueous organic solvents. Carbohydr. Res. 324, 268–274. 10.1016/S0008-6215(99)00263-3 10744335

[B137] Kumar M. Nandi S. K. Kaplan D. L. Mandal B. B. (2017). Localized immunomodulatory silk macrocapsules for islet-like spheroid formation and sustained insulin production. ACS Biomater. Sci. Eng. 3, 2443–2456. 10.1021/acsbiomaterials.7b00218 33445302

[B138] Kuriyama K. Fujiwara A. Inagaki K. Abe Y. (2000). Anti-inflammatory action of a novel peptide, SEK-1005, isolated from a Streptomyces. Eur. J. Pharmacol. 390, 223–228. 10.1016/S0014-2999(00)00017-0 10708727

[B139] Kuwabara R. Hamaguchi M. Fukuda T. Sakai H. Inui M. Sakaguchi S.. (2018). Long-term functioning of allogeneic islets in subcutaneous tissue pretreated with a novel cyclic peptide without immunosuppressive medication. Transplantation 102, 417–425. 10.1097/TP.0000000000001923 28858989

[B140] Lacy P. E. Hegre O. D. Gerasimidi-Vazeou A. Gentile F. T. Dionne K. E. (1991). Maintenance of normoglycemia in diabetic mice by subcutaneous xenografts of encapsulated islets. Science 254, 1782–1784. 10.1126/science.1763328 1763328

[B141] Lahooti S. Sefton M. V. (1999). Methods for microencapsulation with HEMA-MMA. Tissue Eng. Methods Protoc. 18, 331–348. 10.1385/0-89603-516-6:331 21370188

[B142] Lahooti S. Sefton M. V. (2000). Effect of an immobilization matrix and capsule membrane permeability on the viability of encapsulated HEK cells. Biomaterials 21, 987–995. 10.1016/S0142-9612(99)00251-3 10768750

[B143] Laporte C. Tubbs E. Cristante J. Gauchez A.-S. Pesenti S. Lamarche F.. (2019). Human mesenchymal stem cells improve rat islet functionality under cytokine stress with combined upregulation of heme oxygenase-1 and ferritin. Stem Cell Res. Ther. 10, 85–85. 10.1186/s13287-019-1190-4 30867050 PMC6416979

[B144] Lazarjani H. A. Vasheghani-Farahani E. Barani L. Hashemi-Najafabadi S. Shojaosadati S. A. Zahediasl S.. (2010). Effect of polymer concentration on camouflaging of pancreatic islets with mPEG-succinimidyl carbonate. Arti. Cells Blood Substit. Biotechnol. 38, 250–258. 10.3109/10731199.2010.488634 20486872

[B145] Lee C. H. Singla A. Lee Y. (2001). Biomedical applications of collagen. Int. J. Pharm. 221, 1–22. 10.1016/S0378-5173(01)00691-3 11397563

[B146] Li L. Fang Y. Vreeker R. Appelqvist I. Mendes E. (2007). Reexamining the egg-box model in calcium–alginate gels with X-ray diffraction. Biomacromolecules 8, 464–468. 10.1021/bm060550a 17291070

[B147] Li R. H. (1998). Materials for immunoisolated cell transplantation. Adv Drug Deliv Rev 33, 87–109. 10.1016/S0169-409X(98)00022-2 10837655

[B148] Li Y. Fan P. Ding X.-M. Tian X.-H. Feng X.-S. Yan H.. (2017). Polyglycolic acid fibrous scaffold improving endothelial cell coating and vascularization of islet. Chin. Med. J. 130, 832–839. 10.4103/0366-6999.202730 28345548 PMC5381318

[B149] Lin C.-C. Metters A. T. Anseth K. S. (2009). Functional PEG–peptide hydrogels to modulate local inflammation inducedby the pro-inflammatory cytokine TNFα. Biomaterials 30, 4907–4914. 10.1016/j.biomaterials.2009.05.083 19560813 PMC2752207

[B150] Liu W. F. Ma M. Bratlie K. M. Dang T. T. Langer R. Anderson D. G. (2011). Real-time in vivo detection of biomaterial-induced reactive oxygen species. Biomaterials 32, 1796–1801. 10.1016/j.biomaterials.2010.11.029 21146868 PMC4130482

[B151] Llacua A. de Haan B. J. Smink S. A. de Vos P. (2016). Extracellular matrix components supporting human islet function in alginate-based immunoprotective microcapsules for treatment of diabetes. J. Biomed. Mater. Res. A 104, 1788–1796. 10.1002/jbm.a.35706 26990360

[B152] Llacua L. A. de Haan B. J. de Vos P. (2018a). Laminin and collagen IV inclusion in immunoisolating microcapsules reduces cytokine-mediated cell death in human pancreatic islets. J. Tissue Eng. Regen. Med. 12, 460–467. 10.1002/term.2472 28508555

[B153] Llacua L. A. Faas M. M. de Vos P. (2018b). Extracellular matrix molecules and their potential contribution to the function of transplanted pancreatic islets. Diabetologia 61, 1261–1272. 10.1007/s00125-017-4524-8 29306997 PMC6449002

[B154] Llacua L. A. Hoek A. de Haan B. J. de Vos P. (2018c). Collagen type VI interaction improves human islet survival in immunoisolating microcapsules for treatment of diabetes. Islets 10, 60–68. 10.1080/19382014.2017.1420449 29521546 PMC5895175

[B155] Lou S. Zhang X. Zhang J. Deng J. Kong D. Li C. (2017). Pancreatic islet surface bioengineering with a heparin-incorporated starPEG nanofilm. Mater. Sci. Eng. C 78, 24–31. 10.1016/j.msec.2017.03.295 28575981

[B156] Ludwig B. Ludwig S. Steffen A. Knauf Y. Zimerman B. Heinke S.. (2017). Favorable outcome of experimental islet xenotransplantation without immunosuppression in a nonhuman primate model of diabetes. Proc. Natl. Acad. Sci. U.S.A. 114, 11745–11750. 10.1073/pnas.1708420114 29078330 PMC5676906

[B157] Ludwig B. Zimerman B. Steffen A. Yavriants K. Azarov D. Reichel A.. (2010). A novel device for islet transplantation providing immune protection and oxygen supply. Horm. Metab. Res. 42, 918–922. 10.1055/s-0030-1267916 21031332

[B158] Lutolf M. P. Hubbell J. A. (2005). Synthetic biomaterials as instructive extracellular microenvironments for morphogenesis in tissue engineering. Nat. Biotechnol. 23, 47–55. 10.1038/nbt1055 15637621

[B159] Manzoli V. Colter D. C. Dhanaraj S. Fornoni A. Ricordi C. Pileggi A.. (2017). Engineering human renal epithelial cells for transplantation in regenerative medicine. Med. Eng. Phys. 48, 3–13. 10.1016/j.medengphy.2017.03.009 28416198

[B160] Manzoli V. Villa C. Bayer A. L. Morales L. C. Molano R. D. Torrente Y.. (2018). Immunoisolation of murine islet allografts in vascularized sites through conformal coating with polyethylene glycol. Am. J. Transplant. 18, 590–603. 10.1111/ajt.14547 29068143 PMC5820142

[B161] Mao D. Zhu M. Zhang X. Ma R. Yang X. Ke T.. (2017). A macroporous heparin-releasing silk fibroin scaffold improves islet transplantation outcome by promoting islet revascularisation and survival. Acta Biomater. 59, 210–220. 10.1016/j.actbio.2017.06.039 28666883

[B162] Marchioli G. Zellner L. Oliveira C. Engelse M. Koning E. Mano J.. (2017). Layered PEGDA hydrogel for islet of Langerhans encapsulation and improvement of vascularization. J. Mater. Sci. Mater. Med. 28, 195–195. 10.1007/s10856-017-6004-6 29151130 PMC5694514

[B163] Marinucci L. Lilli C. Guerra M. Belcastro S. Becchetti E. Stabellini G.. (2003). Biocompatibility of collagen membranes crosslinked with glutaraldehyde or diphenylphosphoryl azide: an *in vitro* study. J. Biomed. Mater. Res. Part A 67A, 504–509. 10.1002/jbm.a.10082 14566791

[B164] Matsumoto S. Abalovich A. Wechsler C. Wynyard S. Elliott R. B. (2016). Clinical benefit of islet xenotransplantation for the treatment of type 1 diabetes. EBioMedicine 12, 255–262. 10.1016/j.ebiom.2016.08.034 27592597 PMC5078586

[B165] Ménard M. Dusseault J. Langlois G. Baille W. E. Tam S. K. Yahia L.. (2010). Role of protein contaminants in the immunogenicity of alginates. J. Biomed. Mater. Res. B Appl. Biomater. 93, 333–340. 10.1002/jbm.b.31570 20225212

[B166] Michel R. Pasche S. Textor M. Castner D. G. (2005). Influence of PEG architecture on protein adsorption and conformation. Langmuir 21, 12327–12332. 10.1021/la051726h 16343010 PMC2515350

[B167] Miura S. Teramura Y. Iwata H. (2006). Encapsulation of islets with ultra-thin polyion complex membrane through poly(ethylene glycol)-phospholipids anchored to cell membrane. Biomaterials 27, 5828–5835. 10.1016/j.biomaterials.2006.07.039 16919725

[B168] Nair G. G. Liu J. S. Russ H. A. Tran S. Saxton M. S. Chen R.. (2019). Recapitulating endocrine cell clustering in culture promotes maturation of human stem-cell-derived β cells. Nat. Cell Biol. 21, 263–274. 10.1038/s41556-018-0271-4 30710150 PMC6746427

[B169] Najjar M. Manzoli V. Abreu M. Villa C. Martino M. M. Molano R. D.. (2015). Fibrin gels engineered with pro-angiogenic growth factors promote engraftment of pancreatic islets in extrahepatic sites in mice. Biotechnol. Bioeng. 112, 1916–1926. 10.1002/bit.25589 25786390

[B170] Neufeld T. Ludwig B. Barkai U. Weir G. C. Colton C. K. Evron Y.. (2013). The efficacy of an immunoisolating membrane system for islet xenotransplantation in minipigs. PLoS ONE 8:e70150. 10.1371/journal.pone.0070150 23936385 PMC3731363

[B171] Nguyen K. T. West J. L. (2002). Photopolymerizable hydrogels for tissue engineering applications. Biomaterials 23, 4307–4314. 10.1016/S0142-9612(02)00175-8 12219820

[B172] Nuttelman C. R. Rice M. A. Rydholm A. E. Salinas C. N. Shah D. N. Anseth K. S. (2008). Macromolecular monomers for the synthesis of hydrogel niches and their application in cell encapsulation and tissue engineering. Prog. Polym. Sci. 33, 167–179. 10.1016/j.progpolymsci.2007.09.006 19461945 PMC2390836

[B173] Ock S. A. Oh K. B. Hwang S. Yun I. J. Ahn C. Chee H. K.. (2018). Immune molecular profiling of whole blood drawn from a non-human primate cardiac xenograft model treated with anti-CD154 monoclonal antibodies. Xenotransplantation 25:e12392. 10.1111/xen.12392 29582477

[B174] Olabisi R. M. (2015). Cell microencapsulation with synthetic polymers. J. Biomed. Mater. Res. A 103, 846–859. 10.1002/jbm.a.35205 24771675 PMC4309473

[B175] Omami M. McGarrigle J. J. Reedy M. Isa D. Ghani S. Marchese E.. (2017). Islet microencapsulation: strategies and clinical status in diabetes. Curr. Diab. Rep. 17:47. 10.1007/s11892-017-0877-0 28523592

[B176] Orive G. Emerich D. Khademhosseini A. Matsumoto S. Hernández R. M. Pedraz J. L.. (2018). Engineering a clinically translatable bioartificial pancreas to treat type I diabetes. Trends Biotechnol. 36, 445–456. 10.1016/j.tibtech.2018.01.007 29455936

[B177] Orive G. Tam S. K. Pedraz J. L. Hallé J.-P. (2006). Biocompatibility of alginate–poly-l-lysine microcapsules for cell therapy. Biomaterials 27, 3691–3700. 10.1016/j.biomaterials.2006.02.048 16574222

[B178] Ostgaard K. Knutsen S. H. Dyrset N. Aasen I. M. (1993). Production and characterization of guluronate lyase from *Klebsiella pneumoniae* for applications in seaweed biotechnology. Enzyme Microb. Technol. 15, 756–763. 10.1016/0141-0229(93)90006-N 7764007

[B179] O'Sullivan E. S. Vegas A. Anderson D. G. Weir G. C. (2011). Islets transplanted in immunoisolation devices: a review of the progress and the challenges that remain. Endocr. Rev. 32, 827–844. 10.1210/er.2010-0026 21951347 PMC3591674

[B180] Pagliuca F. W. Millman J. R. Gürtler M. Segel M. Van Dervort A. Ryu J. H.. (2014). Generation of functional human pancreatic beta cells *in vitro*. Cell 159, 428–439. 10.1016/j.cell.2014.09.040 25303535 PMC4617632

[B181] Paredes-Juarez A. G. de Haan J. B. Faas M. M. de Vos P. (2014a). A technology platform to test the efficacy of purification of alginate. Materials 7, 2087–2103. 10.3390/ma7032087 28788557 PMC5453257

[B182] Paredes-Juarez G. A. de Haan B. J. Faas M. M. de Vos P. (2013). The role of pathogen-associated molecular patterns in inflammatory responses against alginate based microcapsules. J. Control. Release Official J. Control. Release Soc. 172, 983–992. 10.1016/j.jconrel.2013.09.009 24051034

[B183] Paredes-Juarez G. A. Sahasrabudhe N. M. Tjoelker R. S. de Haan B. J. Engelse M. A. de Koning E. J.. (2015). DAMP production by human islets under low oxygen and nutrients in the presence or absence of an immunoisolating-capsule and necrostatin-1. Sci. Rep. 5:14623. 10.1038/srep14623 26419792 PMC4588515

[B184] Paredes-Juárez G. A. Spasojevic M. Faas M. M. de Vos P. (2014b). Immunological and technical considerations in application of alginate-based microencapsulation systems. Front Bioeng Biotechnol 2:26. 10.3389/fbioe.2014.00026 25147785 PMC4123607

[B185] Park H.-S. Kim J.-W. Lee S.-H. Yang H. K. Ham D.-S. Sun C.-L.. (2017). Antifibrotic effect of rapamycin containing polyethylene glycol-coated alginate microcapsule in islet xenotransplantation. J. Tissue Eng. Regen. Med. 11, 1274–1284. 10.1002/term.2029 26043934

[B186] Pelegrin M. Marin M. Noël D. Del Rio M. Saller R. Stange J.. (1998). Systemic long-term delivery of antibodies in immunocompetent animals using cellulose sulphate capsules containing antibody-producing cells. Gene Ther. 5, 828–834. 10.1038/sj.gt.3300632 9747463

[B187] Perez-Basterrechea M. Esteban M. M. Vega J. A. Obaya A. J. (2018). Tissue-engineering approaches in pancreatic islet transplantation. Biotechnol. Bioeng. 115, 3009–3029. 10.1002/bit.26821 30144310

[B188] Pham T. T. Nguyen T. T. Pathak S. Regmi S. Nguyen H. T. Tran T. H.. (2018). Tissue adhesive FK506–loaded polymeric nanoparticles for multi–layered nano–shielding of pancreatic islets to enhance xenograft survival in a diabetic mouse model. Biomaterials 154, 182–196. 10.1016/j.biomaterials.2017.10.049 29128846

[B189] Phelps E. A. Headen D. M. Taylor W. R. Thulé P. M. García A. J. (2013). Vasculogenic bio-synthetic hydrogel for enhancement of pancreatic islet engraftment and function in type 1 diabetes. Biomaterials 34, 4602–4611. 10.1016/j.biomaterials.2013.03.012 23541111 PMC3628538

[B190] Pişkin E. (1995). Biodegradable polymers as biomaterials. J. Biomater. Sci. Polym. Ed. 6, 775–795. 10.1163/156856295X00175 7772566

[B191] Prochorov A. V. Tretjak S. I. Goranov V. A. Glinnik A. A. Goltsev M. V. (2008). Treatment of insulin dependent diabetes mellitus with intravascular transplantation of pancreatic islet cells without immunosuppressive therapy. Adv. Med. Sci. 53, 240–244. 10.2478/v10039-008-0045-5 19230310

[B192] Prokop A. Wang T. G. (1997). Purification of polymers used for fabrication of an immunoisolation barrier. Ann. N. Y. Acad. Sci. 831, 223–231. 10.1111/j.1749-6632.1997.tb52197.x 9616714

[B193] Ramachandran G. N. (1963). Molecular structure of collagen. Int. Rev. Connect. Tissue Res. 1, 127–182. 10.1016/B978-1-4831-6755-8.50009-7 14110864

[B194] Ren Z. Liu W. Song X. Qi Y. Zhang C. Gao Z.. (2018). Antioxidant and anti-inflammation of enzymatic-hydrolysis residue polysaccharides by Lentinula edodes. Int. J. Biol. Macromol. 120, 811–822. 10.1016/j.ijbiomac.2018.08.114 30145161

[B195] Rengifo H. R. Giraldo J. A. Labrada I. Stabler C. L. (2014). Long-term survival of allograft murine islets coated via covalently stabilized polymers. Adv. Healthcare Mater. 3, 1061–1070. 10.1002/adhm.201300573 24497465 PMC4107175

[B196] Ricordi C. Strom T. B. (2004). Clinical islet transplantation: advances and immunological challenges. Nat. Rev. Immunol. 4, 259–268. 10.1038/nri1332 15057784

[B197] Risbud M. V. Bhargava S. Bhonde R. R. (2003). *In vivo* biocompatibility evaluation of cellulose macrocapsules for islet immunoisolation: implications of low molecular weight cut-off. J. Biomed. Mater. Res. Part A 66A, 86–92. 10.1002/jbm.a.10522 12833434

[B198] Risbud M. V. Bhonde R. R. (2001). Suitability of cellulose molecular dialysis membrane for bioartificial pancreas: *in vitro* biocompatibility studies. J. Biomed. Mater. Res. 54, 436–444. 10.1002/1097-4636(20010305)54:3<436::AID-JBM180>3.0.CO;2-8 11189052

[B199] Robertson R. P. (2004). Islet transplantation as a treatment for diabetes - a work in progress. N. Engl. J. Med. 350, 694–705. 10.1056/NEJMra032425 14960745

[B200] Ronel S. H. D'Andrea M. J. Hashiguchi H. Klomp G. F. Dobelle W. H. (1983). Macroporous hydrogel membranes for a hybrid artificial pancreas. I. Synthesis and chamber fabrication. J. Biomed. Mater. Res. 17, 855–864. 10.1002/jbm.820170512 6619181

[B201] Ruel-Gariépy E. Leclair G. Hildgen P. Gupta A. Leroux J. C. (2002). Thermosensitive chitosan-based hydrogel containing liposomes for the delivery of hydrophilic molecules. J. Control. Release 82, 373–383. 10.1016/S0168-3659(02)00146-3 12175750

[B202] Ryan A. J. O'Neill H. S. Duffy G. P. O'Brien F. J. (2017). Advances in polymeric islet cell encapsulation technologies to limit the foreign body response and provide immunoisolation. Curr. Opin. Pharmacol. 36, 66–71. 10.1016/j.coph.2017.07.013 28865291

[B203] Ryan E. A. Lakey J. R. T. Paty B. W. Imes S. Korbutt G. S. Kneteman N. M.. (2002). Successful islet transplantation continued insulin reserve provides long-term glycemic control. Diabetes 51, 2148–2157. 10.2337/diabetes.51.7.2148 12086945

[B204] Ryan E. A. Paty B. W. Senior P. A. Bigam D. Alfadhli E. Kneteman N. M.. (2005). Five-year follow-up after clinical islet transplantation. Diabetes 54, 2060–2069. 10.2337/diabetes.54.7.2060 15983207

[B205] Sabnis A. Rahimi M. Chapman C. Nguyen K. T. (2009). Cytocompatibility studies of an *in situ* photopolymerized thermoresponsive hydrogel nanoparticle system using human aortic smooth muscle cells. J. Biomed. Mater. Res. Part A 91A, 52–59. 10.1002/jbm.a.32194 PMC2732757 18690661

[B206] Safley S. A. Kenyon N. S. Berman D. M. Barber G. F. Willman M. Duncanson S.. (2018). Microencapsulated adult porcine islets transplanted intraperitoneally in streptozotocin-diabetic non-human primates. Xenotransplantation 25:e12450. 10.1111/xen.12450 30117193

[B207] Schaffellner S. Stadlbauer V. Stiegler P. Hauser O. Halwachs G. Lackner C.. (2005). Porcine islet cells microencapsulated in sodium cellulose sulfate. Transplant. Proc. 37, 248–252. 10.1016/j.transproceed.2005.01.042 15808610

[B208] Schneider S. Feilen P. J. Slotty V. Kampfner D. Preuss S. Berger S.. (2001). Multilayer capsules: a promising microencapsulation system for transplantation of pancreatic islets. Biomaterials 22, 1961–1970. 10.1016/S0142-9612(00)00380-X 11426874

[B209] Sevastianov V. I. Tseytlina E. A. Volkov A. V. Shumakov V. I. (1984). Importance of adsorption-desorption processes of plasma proteins in biomaterials hemocompatibility. ASAIO J. 30, 137–142. 6533878

[B210] Shapiro A. M. Lakey J. R. Ryan E. A. Korbutt G. S. Toth E. Warnock G. L.. (2000). Islet transplantation in seven patients with type 1 diabetes mellitus using a glucocorticoid-free immunosuppressive regimen. N. Engl. J. Med. 343, 230–238. 10.1056/NEJM200007273430401 10911004

[B211] Shin J. S. Kim J. M. Kim J. S. Min B. H. Kim Y. H. Kim H. J.. (2015). Long-term control of diabetes in immunosuppressed nonhuman primates (NHP) by the transplantation of adult porcine islets. Am. J. Transplant. 15, 2837–2850. 10.1111/ajt.13345 26096041

[B212] Shin J. S. Kim J. S. Kim J. M. Jang J. Y. Kim Y. H. Kim H. J.. (2014). Minimizing immunosuppression in islet xenotransplantation. Immunotherapy 6, 419–430. 10.2217/imt.14.14 24815782

[B213] Silva A. I. de Matos A. N. Brons I. G. Mateus M. (2006). An overview on the development of a bio-artificial pancreas as a treatment of insulin-dependent diabetes mellitus. Med. Res. Rev. 26, 181–222. 10.1002/med.20047 16342061

[B214] Sobol M. Bartkowiak A. de Haan B. de Vos P. (2013). Cytotoxicity study of novel water-soluble chitosan derivatives applied as membrane material of alginate microcapsules. J. Biomed. Mater. Res. Part A 101A, 1907–1914. 10.1002/jbm.a.34500 23203606

[B215] Somo S. I. Langert K. Yang C.-Y. Vaicik M. K. Ibarra V. Appel A. A.. (2018). Synthesis and evaluation of dual crosslinked alginate microbeads. Acta Biomater. 65, 53–65. 10.1016/j.actbio.2017.10.046 29101016 PMC5902406

[B216] Sondermeijer H. P. Witkowski P. Woodland D. Seki T. Aangenendt F. J. van der Laarse A.. (2016). Optimization of alginate purification using polyvinylidene difluoride membrane filtration: effects on immunogenicity and biocompatibility of three-dimensional alginate scaffolds. J. Biomater. Appl. 31, 510–520. 10.1177/0885328216645952 27114440 PMC5479495

[B217] Sousa S. G. Oliveira L. A. de Aguiar Magalhães D. de Brito T. V. Batista J. A. Pereira C. M. C.. (2018). Chemical structure and anti-inflammatory effect of polysaccharide extracted from *Morinda citrifolia* Linn (Noni). Carbohydr. Polym. 197, 515–523. 10.1016/j.carbpol.2018.06.042 30007642

[B218] Spasojevic M. Bhujbal S. Paredes G. de Haan B. J. Schouten A. J. de Vos P. (2014a). Considerations in binding diblock copolymers on hydrophilic alginate beads for providing an immunoprotective membrane. J. Biomed. Mater. Res. A 102, 1887–1896. 10.1002/jbm.a.34863 23853069 PMC4232034

[B219] Spasojevic M. Paredes-Juarez G. A. Vorenkamp J. de Haan B. Schouten A. J. de Vos P. (2014b). Reduction of the inflammatory responses against alginate-poly-L-lysine microcapsules by anti-biofouling surfaces of PEG-b-PLL diblock copolymers. PLoS ONE 9:e109837. 10.1371/journal.pone.0109837 25347191 PMC4209974

[B220] Staels W. De Groef S. Heremans Y. Coppens V. Van Gassen N. Leuckx G.. (2016). Accessory cells for β-cell transplantation. Diabetes Obes. Metab. 18, 115–124. 10.1111/dom.12556 26289770

[B221] Stevenson W. T. Sefton M. V. (1987). Graft copolymer emulsions of sodium alginate with hydroxyallsyl methacrylates for microencapsulation. Biomaterials 8, 449–457. 10.1016/0142-9612(87)90081-0 3427143

[B222] Stokke B. T. Smidsroed O. Bruheim P. Skjaak-Braek G. (1991). Distribution of uronate residues in alginate chains in relation to alginate gelling properties. Macromolecules 24, 4637–4645. 10.1021/ma00016a026

[B223] Sugamori M. E. Sefton M. V. (1989). Microencapsulation of pancreatic islets in a water insoluble polyacrylate. ASAIO Trans. 35, 791–799. 2692662

[B224] Surzyn M. Symes J. Medin J. A. Sefton M. V. (2009). IL-10 secretion increases signal persistence of HEMA-MMA-microencapsulated luciferase-modified CHO fibroblasts in mice. Tissue Eng. Part A 15, 127–136. 10.1089/ten.tea.2008.0028 18710337

[B225] Syed F. Bugliani M. Novelli M. Olimpico F. Suleiman M. Marselli L.. (2018). Conformal coating by multilayer nano-encapsulation for the protection of human pancreatic islets: *in-vitro* and *in-vivo* studies. Nanomed. Nanotechnol. Biol. Med. 14, 2191–2203. 10.1016/j.nano.2018.06.013 30016718

[B226] Szymanska E. Winnicka K. (2015). Stability of chitosan-a challenge for pharmaceutical and biomedical applications. Marine Drugs 13, 1819–1846. 10.3390/md13041819 25837983 PMC4413189

[B227] Takemoto N. Konagaya S. Kuwabara R. Iwata H. (2015). Coaggregates of regulatory T cells and islet cells allow long-term graft survival in liver without immunosuppression. Transplantation 99, 942–947. 10.1097/TP.0000000000000579 25651308

[B228] Tam S. K. Dusseault J. Polizu S. Ménard M. Hallé J. P. Yahia L. (2006). Impact of residual contamination on the biofunctional properties of purified alginates used for cell encapsulation. Biomaterials 27, 1296–1305. 10.1016/j.biomaterials.2005.08.027 16154192

[B229] Tan P. L. (2010). Company profile: tissue regeneration for diabetes and neurological diseases at Living Cell Technologies. Regen. Med. 5, 181–187. 10.2217/rme.10.4 20210578

[B230] Tashiro H. Iwata H. Warnock G. L. Takagi T. Machida H. Ikada Y.. (1997). Characterization and transplantation of agarose microencapsulated canine islets of Langerhans. Ann. Transplant. 2, 33–39. 10.1016/S0041-1345(97)01369-9 9869862

[B231] Teramura Y. Iwata H. (2010). Bioartificial pancreas: Microencapsulation and conformal coating of islet of Langerhans. Adv. Drug Delivery Rev. 62, 827–840. 10.1016/j.addr.2010.01.005 20138097

[B232] Teramura Y. Kaneda Y. Iwata H. (2007). Islet-encapsulation in ultra-thin layer-by-layer membranes of poly(vinyl alcohol) anchored to poly(ethylene glycol)–lipids in the cell membrane. Biomaterials 28, 4818–4825. 10.1016/j.biomaterials.2007.07.050 17698188

[B233] Tomei A. A. (2018). Engineering confined and prevascularized sites for islet transplantation. Transplantation 102, 1793–1794. 10.1097/TP.0000000000002290 29794938 PMC6197891

[B234] Tomei A. A. Manzoli V. Fraker C. A. Giraldo J. Velluto D. Najjar M.. (2014). Device design and materials optimization of conformal coating for islets of Langerhans. Proc. Natl. Acad. Sci. U.S.A. 111, 10514–10519. 10.1073/pnas.1402216111 24982192 PMC4115512

[B235] Tun T. Inoue K. Hayashi H. Aung T. Gu Y. J. Doi R.. (1996). A newly developed three-layer agarose microcapsule for a promising biohybrid artificial pancreas: rat to mouse xenotransplantation. Cell Transplant. 5(5, Suppl. 1), S59–S63. 10.1016/0963-6897(96)00042-5 8889234

[B236] Uludag H. De Vos P. Tresco P. A. (2000). Technology of mammalian cell encapsulation. Adv Drug Deliv Rev 42, 29–64. 10.1016/S0169-409X(00)00053-3 10942814

[B237] Unsal I. O. Ginis Z. Pinarli F. A. Albayrak A. Cakal E. Sahin M.. (2015). Comparison of therapeutic characteristics of islet cell transplantation simultaneous with pancreatic mesenchymal stem cell transplantation in rats with type 1 diabetes mellitus. Stem Cell Rev. Rep. 11, 526–532. 10.1007/s12015-014-9563-7 25297071

[B238] Unsworth L. D. Sheardown H. Brash J. L. (2008). Protein-resistant poly(ethylene oxide)-grafted surfaces: chain density-dependent multiple mechanisms of action. Langmuir 24, 1924–1929. 10.1021/la702310t 18217777

[B239] Vågesjö E. Christoffersson G. Waldén T. B. Carlsson P.-O. Essand M. Korsgren O.. (2015). Immunological shielding by induced recruitment of regulatory T-lymphocytes delays rejection of islets transplanted in muscle. Cell Transplant. 24, 263–276. 10.3727/096368914X678535 24480306

[B240] van de Weert M. Hennink W. E. Jiskoot W. (2000). Protein instability in poly(lactic-co-glycolic acid) microparticles. Pharm. Res. 17, 1159–1167. 10.1023/A:1026498209874 11145219

[B241] van Hoogmoed C. G. Busscher H. J. de Vos P. (2003). Fourier transform infrared spectroscopy studies of alginate-PLL capsules with varying compositions. J. Biomed. Mater. Res. A 67, 172–178. 10.1002/jbm.a.10086 14517874

[B242] Vegas A. J. Veiseh O. Doloff J. C. Ma M. Tam H. H. Bratlie K. (2016a). Combinatorial hydrogel library enables identification of materials that mitigate the foreign body response in primates. Nat. Biotechnol. 34, 345–352. 10.1038/nbt.3462 26807527 PMC4904301

[B243] Vegas A. J. Veiseh O. Gurtler M. Millman J. R. Pagliuca F. W. Bader A. R. (2016b). Long-term glycemic control using polymer-encapsulated human stem cell-derived beta cells in immune-competent mice. Nat. Med. 22, 306–311. 10.1038/nm.4030 26808346 PMC4825868

[B244] Veiseh O. Doloff J. C. Ma M. Vegas A. J. Tam H. H. Bader A. R.. (2015). Size- and shape-dependent foreign body immune response to materials implanted in rodents and non-human primates. Nat. Mater. 14, 643–651. 10.1038/nmat4290 25985456 PMC4477281

[B245] Vériter S. Gianello P. Igarashi Y. Beaurin G. Ghyselinck A. Aouassar N.. (2014). Improvement of subcutaneous bioartificial pancreas vascularization and function by coencapsulation of pig islets and mesenchymal stem cells in primates. Cell Transplant. 23, 1349–1364. 10.3727/096368913X663550 23461890

[B246] Villa C. Manzoli V. Abreu M. M. Verheyen C. A. Seskin M. Najjar M.. (2017). Effects of composition of alginate-polyethylene glycol microcapsules and transplant site on encapsulated islet graft outcomes in mice. Transplantation 101, 1025–1035. 10.1097/TP.0000000000001454 27525644 PMC5642344

[B247] Wandrey C. Bartkowiak A. Harding S. E. (2010). “Materials for encapsulation,” in Encapsulation Technologies for Active Food Ingredients and Food Processing, eds Zuidam N. Nedovic V. (New York, NY: Springer), 31–100. 10.1007/978-1-4419-1008-0_3

[B248] Wang J. P. Zhang X. X. Wang X. C. (2011). Preparation, characterization and permeation kinetics description of calcium alginate macro-capsules containing shape-stabilize phase change materials. Renew. Energy 36, 2984–2991. 10.1016/j.renene.2011.03.039

[B249] Wang T. Lacík I. Brissová M. Anilkumar A. V. Prokop A. Hunkeler D.. (1997). An encapsulation system for the immunoisolation of pancreatic islets. Nat. Biotechnol. 15, 358–362. 10.1038/nbt0497-358 9094138

[B250] Weaver J. D. Headen D. M. Hunckler M. D. Coronel M. M. Stabler C. L. García A. J. (2018). Design of a vascularized synthetic poly(ethylene glycol) macroencapsulation device for islet transplantation. Biomaterials 172, 54–65. 10.1016/j.biomaterials.2018.04.047 29715595 PMC5967258

[B251] Weber L. M. Lopez C. G. Anseth K. S. (2009). Effects of PEG hydrogel crosslinking density on protein diffusion and encapsulated islet survival and function. J. Biomed. Mater. Res. A 90, 720–729. 10.1002/jbm.a.32134 18570315 PMC2913724

[B252] Wee S. Gombotz W. R. (1998). Protein release from alginate matrices. Adv. Drug Deliv. Rev. 31, 267–285. 10.1016/S0169-409X(97)00124-5 10837629

[B253] Wilson J. T. Haller C. A. Qu Z. Cui W. Urlam M. K. Chaikof E. L. (2010). Biomolecular surface engineering of pancreatic islets with thrombomodulin. Acta Biomater. 6, 1895–1903. 10.1016/j.actbio.2010.01.027 20102751 PMC2872068

[B254] Wu J. Hu M. Qian Y. W. Hawthorne W. J. Burns H. Liuwantara D.. (2017). *In vivo* costimulation blockade-induced regulatory T cells demonstrate dominant and specific tolerance to porcine islet xenografts. Transplantation 101, 1587–1599. 10.1097/TP.0000000000001482 27653300

[B255] Xu Y. Kim C. S. Saylor D. M. Koo D. (2017). Polymer degradation and drug delivery in PLGA-based drug-polymer applications: a review of experiments and theories. J. Biomed. Mater. Res. B Appl. Biomater. 105, 1692–1716. 10.1002/jbm.b.33648 27098357

[B256] Yamamoto T. Teramura Y. Itagaki T. Arima Y. Iwata H. (2016). Interaction of poly(ethylene glycol)-conjugated phospholipids with supported lipid membranes and their influence on protein adsorption. Sci. Technol. Adv. Mater. 17, 677–684. 10.1080/14686996.2016.1240006 27877914 PMC5101893

[B257] Yang H. K. Ham D.-S. Park H.-S. Rhee M. You Y. H. Kim M. J.. (2016). Long-term efficacy and biocompatibility of encapsulated islet transplantation with chitosan-coated alginate capsules in mice and canine models of diabetes. Transplantation 100, 334–343. 10.1097/TP.0000000000000927 26479281

[B258] Yang K. C. Qi Z. Wu C. C. Shirouza Y. Lin F. H. Yanai G.. (2010). The cytoprotection of chitosan based hydrogels in xenogeneic islet transplantation: An in vivo study in streptozotocin-induced diabetic mouse. Biochem. Biophys. Res. Commun. 393, 818–823. 10.1016/j.bbrc.2010.02.089 20171166

[B259] Yin C. Mien Chia S. Hoon Quek C. Yu H. Zhuo R.-X. Leong K. W.. (2003). Microcapsules with improved mechanical stability for hepatocyte culture. Biomaterials 24, 1771–1780. 10.1016/S0142-9612(02)00580-X 12593959

[B260] Yoshimatsu G. Sakata N. Tsuchiya H. Minowa T. Takemura T. Morita H.. (2015). The co-transplantation of bone marrow derived mesenchymal stem cells reduced inflammation in intramuscular islet transplantation. PLoS ONE 10:e0117561. 10.1371/journal.pone.0117561 25679812 PMC4332659

[B261] Young C. J. Poole-Warren L. A. Martens P. J. (2012). Combining submerged electrospray and UV photopolymerization for production of synthetic hydrogel microspheres for cell encapsulation. Biotechnol. Bioeng. 109, 1561–1570. 10.1002/bit.24430 22234803

[B262] Zhang Y. Gao W. Chen Y. Escajadillo T. Ungerleider J. Fang R. H.. (2017). Self-assembled colloidal gel using cell membrane-coated nanosponges as building blocks. ACS Nano 11, 11923–11930. 10.1021/acsnano.7b06968 29116753 PMC6336496

